# IPr^**(4‑Bp)^—Highly Hindered, Ring Extended N‑Heterocyclic Carbenes

**DOI:** 10.1021/acs.organomet.5c00232

**Published:** 2025-08-08

**Authors:** Yuzhuo Sha, Wenchao Chu, Roger Lalancette, Roman Szostak, Michal Szostak

**Affiliations:** † Department of Chemistry, Rutgers University, 73 Warren Street, Newark, New Jersey 07102, United States; ‡ Department of Chemistry, Wroclaw University, F. Joliot-Curie 14, Wroclaw 50-383, Poland

## Abstract

There is a strong demand for the development of sterically hindered N-heterocyclic carbenes due to their potential to stabilize reactive organometallic species. IPr* (IPr* = 1,3-bis­(2,6-bis­(diphenylmethyl)-4-methylphenyl)­imidazo-2-ylidene) is a highly hindered and sterically flexible ligand, which has found broad utilization in coordination chemistry. Herein, we report the synthesis, structural, and electronic characterization of IPr**^(4‑Bp)^—a class of novel, sterically bulky, and easily accessible N-heterocyclic carbene ligands that bear biphenyl wingtips. Coordination chemistry to Ag­(I), Cu­(I), and Pd­(II) is presented. The biphenyl wingtip permits extension of the %V_bur_ of IPr**^(4‑Bp)^ to 58.8%, linear geometry, which is the highest reported to date for imidazol-2-ylidene ligands. The synthesis of an electron-rich congener, IPr**^MeO(4‑Bp)^, which is analogous to the popular IPr*^MeO^ ligand, is also presented. Coordination to Pd­(II) demonstrates steric flexibility, where the %V_bur_ changes to 42.8% for square planar geometry. The study demonstrates that IPr^**(4‑Bp)^ with an extended biphenyl wingtip is sterically bulkier than common imidazol-2-ylidenes, including IPr, IPr*, IPr^#^, and IPr*^(2‑Np)^. Considering the essential role of sterically hindered N-heterocyclic carbene ligands in various areas of coordination chemistry and metal catalysis, this new class of NHCs is poised for rapid and widespread application.

## Graphical abstract

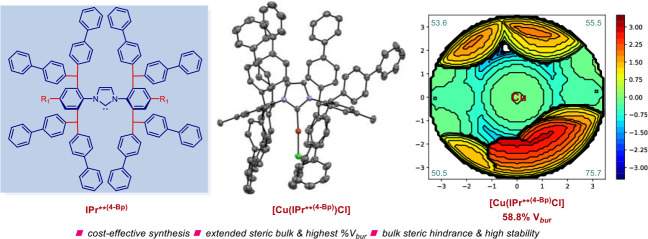

## Introduction

Since the first isolation of N-heterocyclic carbenes by Arduengo in 1991, a plethora of structurally diverse N-heterocyclic carbenes have been developed, and this class of compounds has become widely used as tremendously valuable ligands in the stabilization of reactive metal centers.^1,2^ In particular, the adaptable electronic properties of the carbene center^3^ in conjunction with the variable steric bulk of wingtip groups^4^ permit N-heterocyclic carbene ligands to stabilize metals at various oxidation states as well as precisely define the shape of catalytic pockets, governing the rate and selectivity of catalytic processes.^5,6^ To date, many classes of diverse N-heterocyclic carbene ligands with an assortment of substitutions have been developed.^7^ It has been well-recognized that the most successful are N-heterocyclic carbene ligands that feature significant steric hindrance around the metal center in addition to steric flexibility, enabling the stabilization of organometallic species and the steric adjustment of the metal coordination sphere. In this context, IPr* (IPr* = 1,3-bis­(2,6-bis­(diphenylmethyl)-4-methylphenyl)­imidazo-2-ylidene) is a golden standard of highly hindered and sterically flexible N-heterocyclic carbene ligands that has found broad application in stabilizing reactive metal centers and exhibits universal applicability across many fields. IPr* represents a second generation of classical sterically demanding imidazol-2-ylidene ligands, such as IPr (IPr = 1,3-bis­(2,6-diisopropylphenyl)­imidazol-2-ylidene), which historically have been the first choice of ligands to facilitate challenging bond-forming reactions in organometallic catalysis. The stabilization of reactive metal species through the wingtip steric hindrance of IPr has led to the discovery of IPr*, in which the methyl groups are replaced by phenyl rings, which has resulted in significant advancements in this field.^8^ Related are N-heterocyclic carbenes that feature peralkylation of the N-aromatic wingtips, such as IPr^#^ introduced by our group, which possess a bulkier buried volume compared to IPr and IPr*.^9^ Furthermore, related is IPr*^(2‑Np)^ reported by Marko featuring 2-naphthyl wingtip substitution (Figure 1).^10,11^

**Figure 1:**
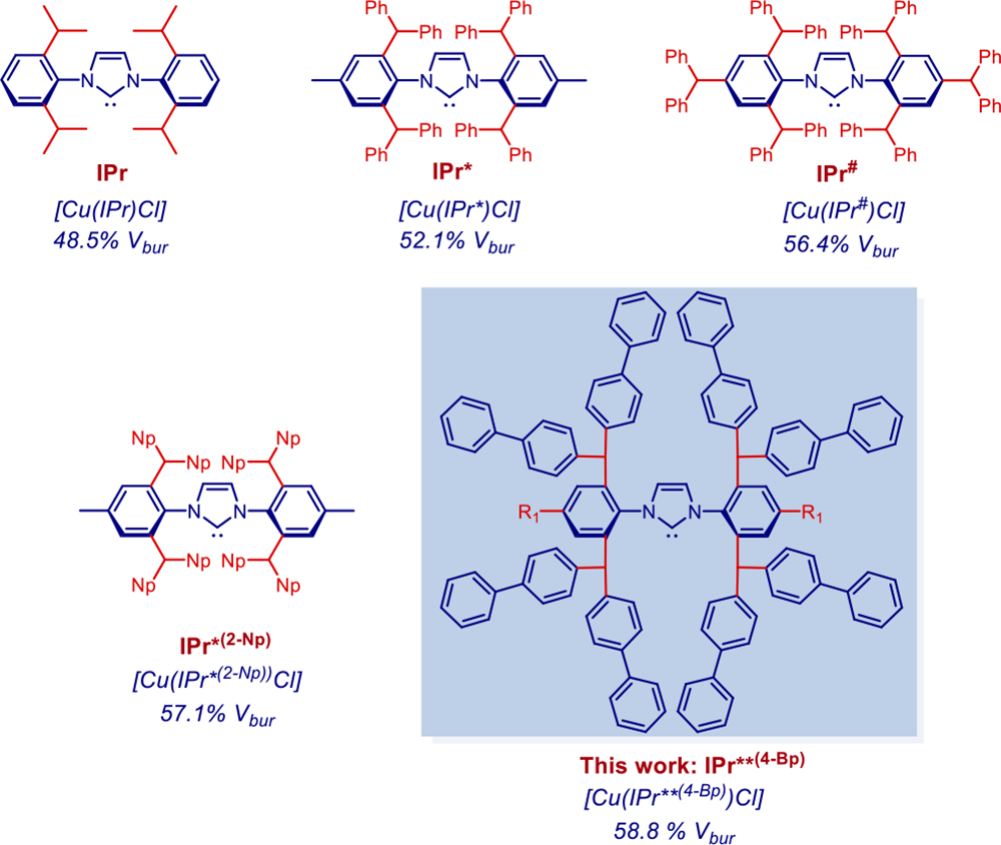
State-of-the-art of significantly sterically demanding N-heterocyclic carbene ligands.

Inspired by our continued interest in N-heterocyclic carbenes and ligand development,^9^ we aimed to expand the concept of sterically hindered, flexible, and easily accessible N-heterocyclic carbene ligands. We envisioned a bulkier variant of IPr, IPr*, IPr^#^, and IPr*^(2‑Np)^ ligands, where the presence of a versatile biphenyl ring group extends beyond the metal coordination sphere and provides greater steric hindrance while retaining full flexibility around the metal center. Herein, we report the synthesis and structural and electronic characterization of IPr**^(4‑Bp)^—a class of novel, sterically bulky, and easily accessible N-heterocyclic carbene ligands that feature two phenyl wingtips, each substituted with two di­(biphenyl)­methyl groups. These substituents are attached via methyl linkages at the 2- and 6-positions of the phenyl rings. Significantly, these IPr**^(4‑Bp)^ ligands extend well beyond the metal coordination sphere (IPr**^(4‑Bp)^ = 1,3-bis­(2,6-bis­(di­([1,1′-biphenyl]-4-yl)­methyl)-4-methyl phenyl imidazol-2-ylidene). Coordination chemistry to Ag­(I), Cu­(I), and Pd­(II) is presented. Structural and electronic characterization indicate that these ligands are more sterically demanding than the classical imidazol-2-ylidenes, such as IPr, IPr*, IPr^#^, and IPr*^(2‑Np)^. The %V_bur_ of IPr**^(4‑Bp)^ of 58.8%, linear geometry, is the highest reported to date for imidazol-2-ylidene ligands. The synthesis of an electron-rich congener, IPr**^MeO(4‑Bp)^, analogous to the popular IPr*^MeO^ ligand, is also presented. Considering the essential role of IPr* in various areas of coordination chemistry, this new class of NHCs is poised for rapid and widespread adoption.

## Results and Discussion

At the outset, IPr**^(4‑Bp)^ and IPr**^MeO(4‑Bp)^ (Bp = biphenyl) were selected as ring-extended structural analogues of the bulky IPr, IPr*, IPr^#^, and IPr*^(2‑Np)^ imidazol-2-ylidene N-heterocyclic carbenes. The synthesis of IPr**^(4‑Bp)^ and IPr**^MeO (4‑Bp)^ is outlined in Scheme 1. The synthesis involves three cost-effective and operationally simple synthetic steps. Commercially available 4-bromo-1,1’-biphenyl (**1**) was converted into di­([1,1′-biphenyl]-4-yl)­methanol (**2**) using Grignard addition to ethyl formate in 87% yield. Next, 2,6-bis-biphenylanilines (**3**) and (**4**) were prepared in 75 and 79% yield by Friedel–Crafts benzylation in the presence of HCl (1.0 equiv) and ZnCl_2_ (0.5 equiv) at 160 °C. The synthesis of final ligands IPr**^(4‑Bp)^·HCl (**5**) and IPr**^MeO(4‑Bp)^·HCl (**6**) was successfully achieved by reacting anilines with glyoxal (1.1 equiv) and (CH_2_O)_*n*_ (0.9 equiv) in the presence of HCl (1.1 equiv) in CHCl_3_ at 60 °C in 65 and 72% yield. The desired imidazolium salts were readily obtained on a gram scale by trituration with diethyl ether, thus obviating the need for chromatographic purification. The same route can be used for the synthesis of both IPr**^(4‑Bp)^ and IPr**^MeO (4‑Bp)^ salts, highlighting the practicality of the approach.

**Scheme 1:**
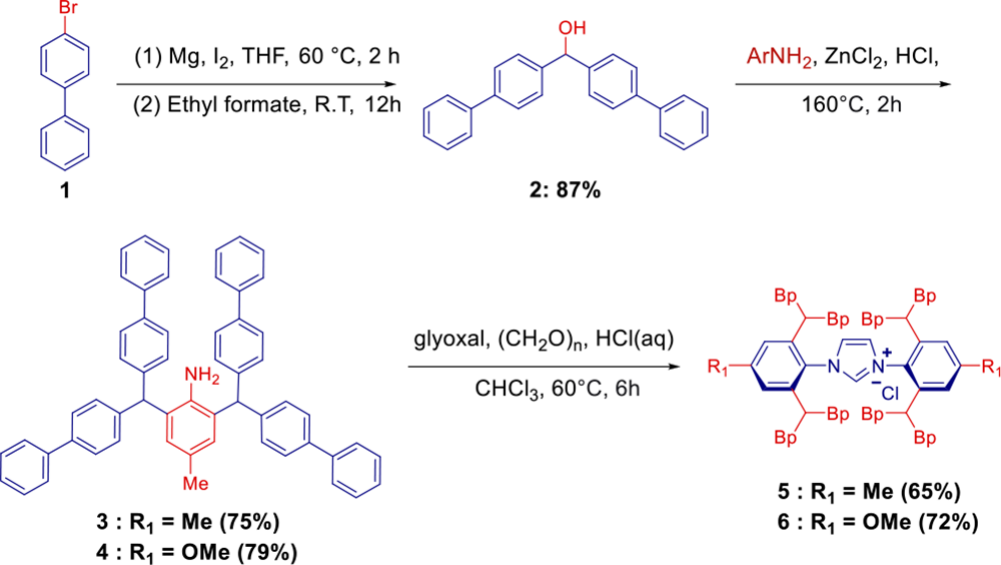
Synthesis of IPr**^(4‑Bp)^·HCl and IPr**^MeO(4‑Bp)^·HCl^a^

a Conditions: (a) 1) 4-bromo-1,1′-biphenyl, (2.0 equiv), Mg (1.0 equiv), I_2_, THF, 60 °C, 2 h; 2) HCO_2_Et, (1.0 equiv), THF, 0 °C then RT, 12 h, 87%; (b) aniline (0.5 equiv), di­([1,1′-biphenyl]-4-yl)­methanol (1.0 equiv), ZnCl_2_ (0.5 equiv), HCl_(aq)_ (0.5 equiv), 160 °C, 30 min, 75%; (c) 40% glyoxal_(aq)_ (1.1 equiv), (CH_2_O)_*n*_ (0.9 equiv), HCl_(aq)_ (1.1 equiv), CHCl_3_, 60 °C, 6 h, 75%.

With access to IPr**^(4‑Bp)^ and IPr**^MeO(4‑Bp)^ salts secured, we next focused on evaluating coordination to Ag­(I), Cu­(I), and Pd­(II). As shown in Scheme 2, silver­(I) complexes were prepared by directly reacting imidazolium salt with Ag_2_O in CH_2_Cl_2_ to afford the Ag­(I)–NHC complex in 95% yield for [Ag­(IPr**^(4‑Bp)^)­Cl] (**7**) and 88% for [Ag­(IPr**^MeO(4‑Bp)^)­Cl] (**8**). Next, [Cu­(IPr**^(4‑Bp)^)­Cl] (**9**) was prepared by generating the free carbene in situ by deprotonation with KO*t*-Bu in THF in 55% yield. Using the same method, [Cu­(IPr**^MeO(4‑Bp)^)­Cl] (**10**) was prepared in 48% yield, while [Pd­(IPr**^(4‑Bp)^)­(3-Cl-py)] (**11**) and [Pd­(IPr**^MeO(4‑Bp)^)­(3-Cl-py)] (**12**) were synthesized using K_2_CO_3_ in the presence of PdCl_2_ and 3-Cl-py at 80 °C. Notably, all complexes [Ag­(IPr**^(4‑Bp)^)­Cl], [Cu­(IPr**^(4‑Bp)^)­Cl], and [Pd­(IPr**^(4‑Bp)^)­(3-Cl-py)­Cl_2_] were found to be stable to air and moisture.

**Scheme 2:**
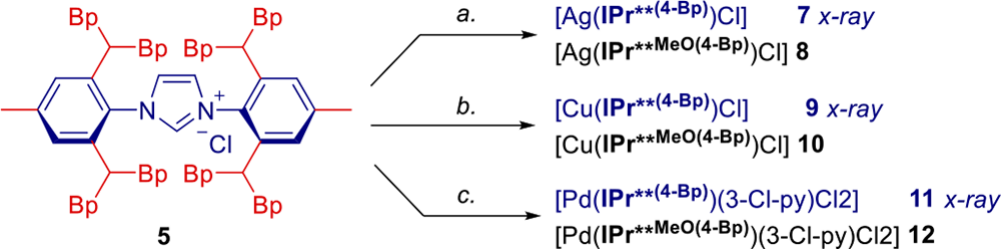
Synthesis of IPr**^(4‑Bp)^ and IPr**^MeO(4‑Bp)^ Complexes^a^

a Conditions: (a) Ag_2_O, CH_2_Cl_2_, RT, overnight, (**7**): 95%, (**8**): 88%; (b) CuCl, *t*BuOK, THF, RT, overnight, (**9**): 55%, (**10**): 48%; (c) PdCl_2_, 3-Cl-Pyridine, K_2_CO_3_, 80 °C, 16 h, (**11**): 85%, (**12**): 70%.

The Ag­(I)–IPr**^(4‑Bp)^, Cu­(I)–IPr**^(4‑Bp)^, and Pd­(II)–IPr**^(4‑Bp)^ complexes were characterized by X-ray crystallography (Figures 2, 3, and 4).^12^ Previous studies by Cavallo, Nolan and co-workers demonstrated that the combined use of the % buried volume (%V_bur_) and steric maps of linear complexes is the best indication for quantifying the steric impact of NHC ligands.^4,13^ As shown, [Ag­(IPr**^(4‑Bp)^)­Cl] and [Cu­(IPr**^(4‑Bp)^)­Cl] are linear (**7**: C–Ag–Cl, 179.16°; C–Ag, 2.091 Å; **9**: C–Cu–Cl, 174.09°; C–Cu, 1.88 Å). In contrast, [Pd­(IPr**^(4‑Bp)^)­(3-Cl-py)­Cl_2_] is characterized by a square planar geometry (**11**: C–Pd–Cl, 89.8°; C–Pd, 1.965 Å).

**Figure 2:**
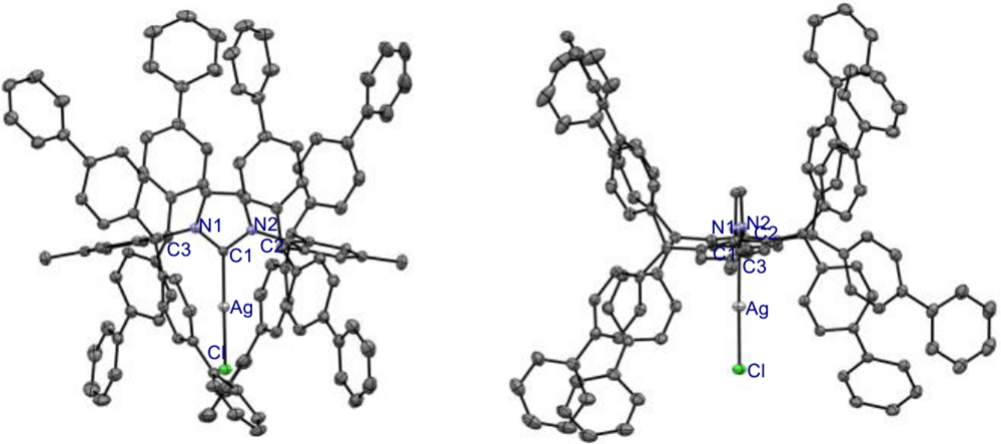
X-ray crystal structure of the [Ag­(IPr**^(4‑Bp)^)­Cl] complex (**7**). Two views: front (left); side (right). Hydrogen atoms have been omitted for clarity. Selected bond lengths [Å] and angles [°]: Ag–Cl, 2.3369; Ag–C1, 2.091; C1–N1, 1.350; C1–N2, 1.349; N1–C3, 1.439; N2–C2, 1.443; C1–Ag–Cl, 179.16; N1–C1–N2, 104.53; C1–N1–C3, 126.23; C1–N2–C2, 126.03. CCDC 2429423. See SI for expanded ORTEP structures.

**Figure 3:**
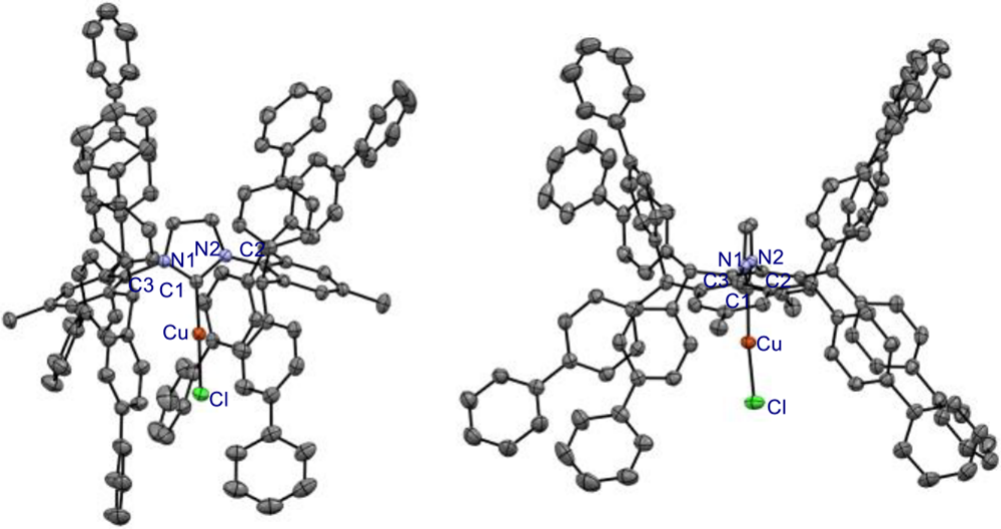
X-ray crystal structure of the [Cu­(IPr**^(4‑Bp)^)­Cl] complex (**9**). Two views: front (left); side (right). Hydrogen atoms have been omitted for clarity. Selected bond lengths [Å] and angles [°]: Cu–Cl, 2.107; Cu–C1, 1.880; C1–N1, 1.361; C1–N2, 1.360; N2–C3, 1.442; N1–C2, 1.446; C1–Cu–Cl, 174.09; N1–C1–N2, 103.67; C1–N1–C2, 123.86; C1–N2–C3, 123.07. CCDC 2429653. See SI for expanded ORTEP structures.

**Figure 4:**
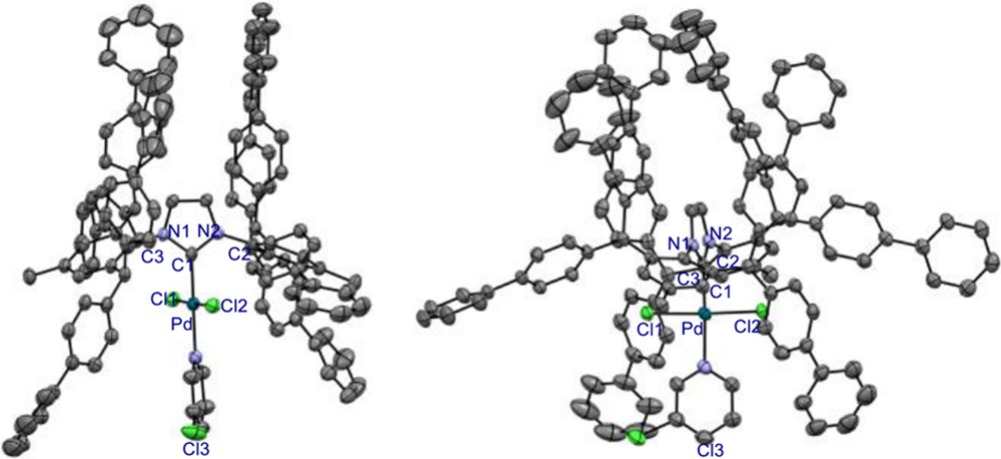
X-ray crystal structure of the [Pd­(IPr**^(4‑Bp)^)­(3-Cl-py)­Cl_2_] complex (**11**). Two views: front (left); side (right). Hydrogen atoms have been omitted for clarity. Selected bond lengths [Å] and angles [°]: Pd1–C1, 1.965; Pd–N3, 2.119; N2–C1, 1.362; N1–C1, 1.359; N1–C3, 1.448; N2–C2, 1.448; C1–Pd–N3, 178.01; N1–C1–N2, 104.96; C1–N2–C2, 124.46; C1–N1–C3, 123.47. CCDC 2429424. See SI for expanded ORTEP structures.

Notably, the (%V_bur_) of [Cu­(IPr**^(4‑Bp)^)­Cl] is 58.8%, which can be compared with the corresponding imidazol-2-ylidene complexes, [Cu­(IPr)­Cl], [Cu­(IPr*)­Cl], [Cu­(IPr^#^)­Cl], and [Cu­(IPr*^(2‑Np)^)­Cl], which are characterized by the increasing (%V_bur_) in the order of 48.5, 52.1, 56.4, and 57.1%, respectively. To our knowledge, the (%V_bur_) of [Cu­(IPr**^(4‑Bp)^)­Cl] is the highest reported to date for simple imidazol-2-ylidene ligands, indicating an enhancement of the steric contribution of the aromatic N-wingtip by the biphenyl substitution.

The (%V_bur_) of [Ag­(IPr**^(4‑Bp)^)­Cl] is 56.2%, which can be compared with the corresponding Ag­(I)–NHC complexes of [Ag­(IPr)­Cl], [Ag­(IPr*)­Cl], [Ag­(IPr^#^)­Cl], and [Ag­(IPr*^(2‑Np)^)­Cl] in the increasing order of 42.8, 53.5, 53.9, and 57.4%, respectively. It is worthwhile to note that the (%V_bur_) value of the naphthyl complex, [Ag­(IPr^*(2‑Np)^)­Cl] (57.4%), is slightly higher than that of [Ag­(IPr**^(4‑Bp)^)­Cl]. In terms of steric distribution, [Ag­(IPr**^(4‑Bp)^)­Cl] extends 4.519 Å from the farthest carbon to the metal plane, which is significantly further away than 3.266 Å of [Ag­(IPr^*(2‑Np)^)­Cl], indicating that IPr**^(4‑Bp)^ provides additional steric hindrance beyond the coordination sphere compared with IPr*^(2‑Np)^ (Figure 5). Furthermore, the (%V_bur_) value of [Pd­(IPr**^(4‑Bp)^)­(3-Cl-py)­Cl_2_] is 42.8%, indicating the capacity of IPr**^(4‑Bp)^ to accommodate bulkier ligands (Figure 6). This observed change in flexibility (%V_bur_ from 58.8 to 42.8%) could be particularly important in catalytic applications, including facilitating reductive elimination in cross-coupling. This steric adjustment indicates that IPr**^(4‑Bp)^ can alter its surroundings by at least 28%, which compares favorably with steric flexibility of IPr*.^8a^

**Figure 5:**
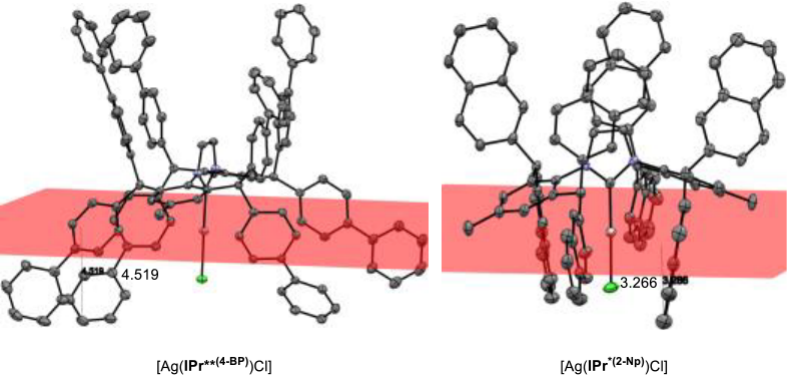
Graphical representation of extended steric demand beyond the metal coordination sphere of biphenyl complex [Ag­(IPr**^(4‑Bp)^)­Cl] (**7**) in comparison with [Ag­(IPr^*(2‑Np)^)­Cl]. Note the 4.519 vs 3.266 Å distance from the metal plane to the farthest away carbon. Hydrogen atoms have been omitted for clarity.

**Figure 6:**
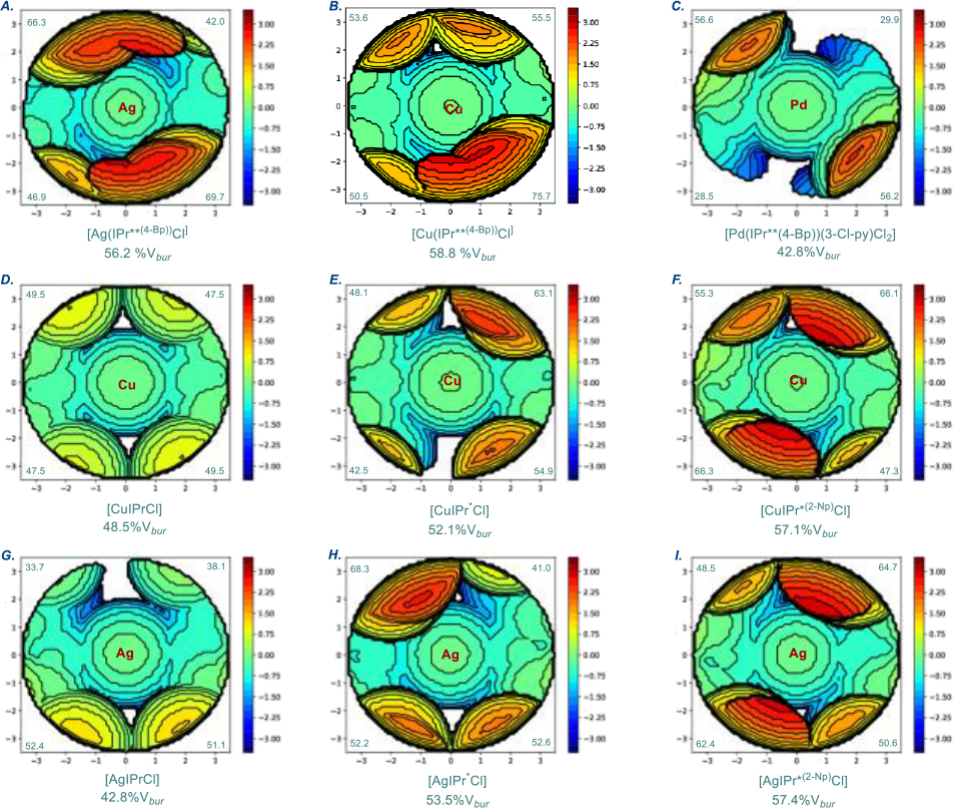
Topographical steric maps of (A) [Ag­(IPr**^(4‑Bp)^)­Cl] (**7**), (B) [Cu­(IPr**^(4‑Bp)^)­Cl] (**9**), (C) [Pd­(IPr**^(4‑Bp)^)­(3-Cl-py)­Cl_2_] (**11**), (D) [Cu­(IPr)­Cl], (E) [Cu­(IPr*)­Cl], (F) [Cu­(IPr*^(2‑Np)^)­Cl], (G) [Ag­(IPr)­Cl], (H) [Ag­(IPr*)­Cl], and (I) [Ag­(IPr*^(2‑Np)^)­Cl], showing % V_bur_ per quadrant.

Importantly, crystallographic analysis of [Ag­(IPr**^(4‑Bp)^)­Cl], [Cu­(IPr**^(4‑Bp)^)­Cl], and [Pd­(IPr**^(4‑Bp)^)­(3-Cl-py)­Cl_2_] reveals spatially distinct, unsymmetrical biaryl quadrant distribution in their respective steric maps ([Ag­(IPr**^(4‑Bp)^)­Cl]: SW 46.9%, NW 66.3%, NE 42.0%, SE 69.7%; [Cu­(IPr**^(4‑Bp)^)­Cl]: SW 50.5%, NW 53.6%, NE 55.5%, SE 75.7%; [Pd­(IPr**^(4‑Bp)^)­(3-Cl-py)­Cl_2_]: SW 28.5%, NW 56.6%, NE 29.9%, SE 56.2%, for each quadrant) (Figures 6A–C). These values indicate further enhancement of the steric impact of IPr**^(4‑Bp)^ as compared to the classical imidazol-2-ylidnes, such as IPr, IPr*, IPr^#^, and IPr*^(2‑Np)^, as indicated by the quadrant distribution in steric maps ([Cu­(IPr)­Cl]: SW 47.5%, NW 49.5%, NE 47.5%, SE 49.5%; [Cu­(IPr*)­Cl]: SW 42.5%, NW 48.1%, NE 63.1%, SE 54.9%; [Cu­(IPr*^(2‑Np)^)­Cl: SW 66.3%, NW 55.3%, NE 66.1%, SE 47.3% (Figures 6D–F). The steric enhancement of IPr**^(4‑Bp)^ is further verified by comparison of the crystallographic data of [Ag­(NHC)­Cl] complexes ([Ag­(IPr)­Cl]: SW 52.4%, NW 33.7%, NE 38.1%, SE 51.1%; [Ag­(IPr*)­Cl]: SW 52.2%, NW 68.3%, NE 41.0%, SE 52.6%; [Ag­(IPr*^(2‑Np)^)­Cl]: SW 62.4%, NW 48.5%, NE 64.7%, SE 50.6%) (Figures 6G–I). Isolation of free carbene has not been performed. At this point, the synthesis of Rh­(I) complexes has not been performed. Ongoing studies are focused on synthetic applications, including group 9 metal complexes, and these results will be reported in due course.

To gain insight into the electronic properties of these wingtip biphenyl N-heterocyclic carbenes, HOMO and LUMO energy levels were determined at the B3LYP 6-311++g­(d,p) level (Figure 7). The σ-donor orbital of IPr**^(4‑Bp)^ (HOMO, −6.03 eV) is slightly higher than that of the sterically hindered IPr* (−6.12 eV) and can be compared with that of the classical IPr (−6.01 eV). Furthermore, the π-accepting orbital of IPr**^(4‑Bp)^ (LUMO+2 due to required symmetry, −1.21 eV) can be compared with those of the sterically hindered IPr* (−0.90 eV) and the classical IPr (−0.48 eV). The LUMO orbital of IPr**^(4‑Bp)^ (−1.26 eV) is located on the biphenyl fragment, as expected. Furthermore, the π-donating orbital of IPr**^(4‑Bp)^ (HOMO–1, −6.09 eV) can be compared with those of the sterically hindered IPr* (−6.28 eV) and the parent IPr (−6.55 eV). Thus, these wingtip biphenyl NHC ligands can be defined as strongly σ-donating carbenes, which is characteristic of imidazol-2-ylidenes, with the distinguishing feature of a very high steric hindrance and extension of the hindrance toward the metal coordination sphere, making them unique from other classes of sterically hindered imidazol-2-ylidenes.

**Figure 7:**
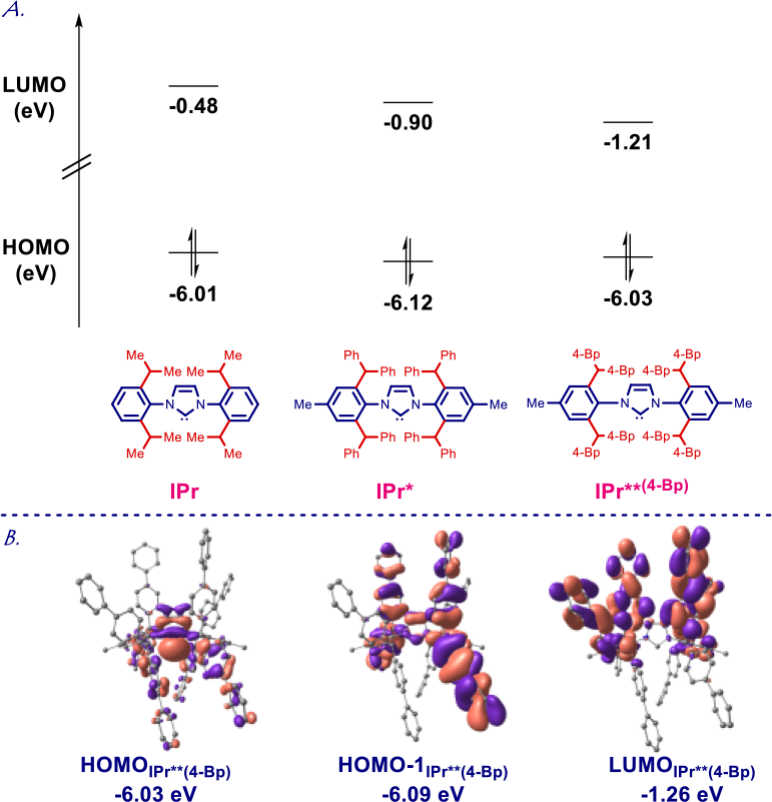
(A) HOMO and LUMO energy levels (eV) of IPr, IPr*, and IPr**^(4‑Bp)^. (B) HOMO, HOMO–1, and LUMO (eV) of IPr**^(4‑Bp)^ calculated at B3LYP 6-311++g­(d,p). See the SI for details.

## Conclusions

In summary, we have reported a new class of sterically bulky, ring-extended N-heterocyclic carbenes that feature a versatile biphenyl wingtip, which reaches beyond the metal coordination sphere. These ligands are easily prepared in three synthetic steps and expand the family of sterically hindered imidazol-2-ylidenes, such as IPr, IPr*, IPr^#^, and IPr*^(2‑Np)^. Coordination to Ag­(I), Cu­(I), and Pd­(II) was presented. Crystallographic analysis provided insights into the steric impact of the biphenyl wingtip, indicating that IPr**^(4‑Bp)^ is the largest simple imidazol-2-ylidene prepared to date. The biphenyl ring extension provides a deep cavity around the metal center through the extended N-aryl wingtip compared with other sterically demanding yet flexible imidazol-2-ylidenes. Coordination to Pd demonstrated significant steric flexibility, where the %V_bur_ can be adjusted by at least one-third to accommodate more bulky ancillary ligands. In view of the major demand for sterically demanding N-heterocyclic carbenes and the extreme steric bulk and flexibility of IPr**^(4‑Bp)^, we believe that this class of NHCs will offer new avenues for stabilization of reactive metal centers in inorganic and organometallic chemistry.

## Supplementary Material





## Notes

All synthetic procedures, NMR characterization of catalyst synthesis, crystallographic details, and computational details (PDF)
Cartesian coordinates with zero-point energies and thermal corrections (XYZ)
