# Supplementary material for: IPr**(4‑Bp)—Highly Hindered, Ring Extended N‑Heterocyclic Carbenes
Source: Organometallics. 2025 Aug 8;44(16):1848–53. doi: 10.1021/acs.organomet.5c00232 (PMC12360280; doi:10.1021/acs.organomet.5c00232)
Supplement: Supplementary file 1 [file om5c00232_si_001.pdf]

Supporting Information

IPr**$^{(4-Bp)}$ – Highly Hindered, Ring Extended N-Heterocyclic Carbenes

Yuzhuo Sha,[†] Wenchao Chu,[*,†] Roger Lalancette,[†] Roman Szostak,[‡] and Michal Szostak[*,†]

[†]Department of Chemistry, Rutgers University, 73 Warren Street, Newark, New Jersey 07102, United States
[‡]Department of Chemistry, Wroclaw University, F. Joliot-Curie 14, Wroclaw 50-383, Poland

michal.szostak@rutgers.edu
wc558@scarletmail.rutgers.edu

Table of Contents S1

General Information S2
General Procedure for the Synthesis of IPr**$^{(4-Bp)}$•HCl S3
General Procedure for the Synthesis of IPr**$^{(4-Bp)}$ Complexes S6
Crystallographic Details S11
Computational Details S17
^{1}H and ^{13}C NMR Spectra S19
References S30

List of Known Compounds/General Methods

All starting materials reported in the manuscript have been previously described in literature and prepared by the method reported previously unless stated otherwise. All experiments were performed using standard Schlenk techniques under nitrogen or argon unless stated otherwise. All solvents were purchased at the highest commercial grade and used as received or after purification by passing through activated alumina columns or distillation from sodium/benzophenone under nitrogen. All solvents were deoxygenated prior to use. All other chemicals were purchased at the highest commercial grade and used as received. Reaction glassware was oven-dried at 140 °C for at least 24 h or flame-dried prior to use, allowed to cool under vacuum and purged with argon (three cycles). All products were identified using ^{1}H NMR analysis and comparison with authentic samples. GC and/or GC/MS analysis was used for volatile products. All yields refer to yields determined by ^{1}H NMR and/or GC or GC/MS using an internal standard (optimization) and isolated yields (preparative runs) unless stated otherwise. ^{1}H NMR and ^{13}C NMR spectra were recorded in $CDCl_3$ on Bruker spectrometers at 500 (^{1}H NMR) and 125 MHz (^{13}C NMR). All shifts are reported in parts per million (ppm) relative to residual $CHCl_3$ peak (7.26 and 77.2 ppm, ^{1}H NMR and ^{13}C NMR, respectively). All coupling constants (*J*) are reported in hertz (Hz). Abbreviations are: s, singlet; d, doublet; t, triplet; q, quartet; brs, broad singlet. GC-MS chromatography was performed using Agilent HP6890 GC System and Agilent 5973A inert XL EI/CI MSD using helium as the carrier gas at a flow rate of 1 mL/min and an initial oven temperature of 50 °C. The injector temperature was 250 °C. The detector temperature was 250 °C. For runs with the initial oven temperature of 50 °C, temperature was increased with a 10 °C/min ramp after 50 °C hold for 3 min to a final temperature of 220 °C, then hold at 220 °C for 15 min (splitless mode of injection, total run time of 22.0 min). High-resolution mass spectra (HRMS) were measured on a 7T Bruker Daltonics FT-MS instrument. All flash chromatography was performed using silica gel, 60 Å, 300 mesh. TLC analysis was carried out on glass plates coated with silica gel 60 F254, 0.2 mm thickness. The plates were visualized using a 254 nm UV lamp or aqueous potassium permanganate. ^{1}H NMR and ^{13}C NMR data are given for all compounds in the Supporting Experimental for characterization purposes. ^{1}H NMR, ^{13}C NMR, and HRMS data are given for all new compounds. All products have been previously reported, unless stated otherwise.

Experimental Procedures and Characterization Data

1. General Procedure for the Synthesis of IPr**(4-Bp)•HCl

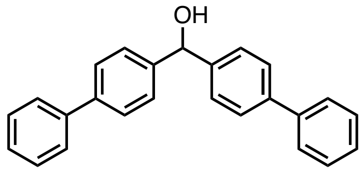

Di([1,1'-biphenyl]-4-yl) methanol (2)

Activated magnesium turnings (1.32 g, 55 mmol, 2.2 equiv) and a few drops of dibromoethane was suspended in anhydrous THF (50 mL). To the mixture at ambient temperature was slowly added a solution of 4-bromo-1,1'-biphenyl (11.7 g, 50 mmol, 2.0 equiv) in anhydrous THF (50 mL). After complete addition, the reaction mixture was heated at 60 °C for 2 h. After indicated time, the mixture was cooled down to room temperature. Ethyl formate (1.85g, 25 mmol, 1 equiv) was slowly added into the above Grignard solution over 10 minutes in ice bath. The resultant brown solution was stirred at room temperature for 12 h, after which the mixture was poured over aqueous NH_4Cl solution. The aqueous layer was extracted with ethyl acetate, and the combined organic extracts were washed with brine and dried over anhydrous Na_2SO_4, filtered and concentrated in vacuo to give white solid as product (yield 87%, 7.4 g). ^{1}H NMR (500 MHz, $CDCl_3$) δ 7.65 – 7.59 (m, 8H), 7.53 (d, J = 7.9 Hz, 4H), 7.46 (t, J = 7.5 Hz, 4H), 7.37 (t, J = 7.4 Hz, 2H), 5.98 (s, 1H), 2.30 (s, 1H). ^{13}C NMR (125 MHz, $CDCl_3$) δ 142.79, 140.79, 140.63, 128.79, 127.36, 127.34, 127.12, 127.00, 75.90. NMR spectroscopic data agreed with literature values.[1]

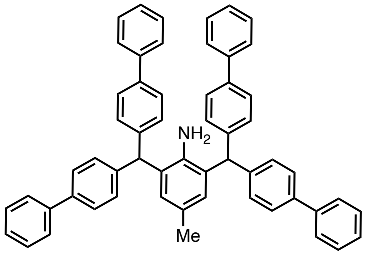

2,6-bis(Di([1,1'-biphenyl]-4-yl) methyl)-4-methylaniline (3)

An oven-dried 250 mL round-bottomed flask equipped with a stir bar was charged with mixture of p-toluidine (1.44 g, 13.5 mmol, 1.0 equiv), di([1,1'-biphenyl]-4-yl) methanol (9.08 g, 27.0 mmol, 2.0 equiv) and anhydrous zinc chloride (0.92 g, 6.8 mmol, 0.5 equiv) in concentrated hydrochloric acid (1.13 mL, 37% in H_2O, 1.0 equiv). The mixture was heated to 160 °C and melted together. After 30 min at 160 °C, the reaction mixture started solidifying. The reaction mixture

was cooled down to room temperature and dissolved in CH_2Cl_2 (100 mL). The CH_2Cl_2 layer was washed with water (3×100 mL) and NH_4Cl aqueous and dried over anhydrous Na_2SO_4. The solution was concentrated to 20 mL. The product was crashed out with 200 ml methanol and washed with methanol (3 × 100 mL). The desired aniline was obtained as a white crystalline solid at 75.0 % (7.53 g) yield. ^{1}H NMR (500 MHz, $CDCl_3$) δ 7.64 – 7.61 (m, 8H), 7.58 (dd, J = 8.2, 1.7 Hz, 8H), 7.49 – 7.42 (m, 8H), 7.36 (td, J = 7.2, 1.2 Hz, 4H), 7.27 – 7.24 (m, 8H), 6.53 (s, 2H), 5.60 (s, 2H), 3.45 (s, 2H), 2.12 (s, 3H). ^{13}C NMR (125 MHz, $CDCl_3$) δ 141.82, 140.77, 139.68, 139.48, 129.97, 129.32, 129.16, 128.57, 127.27, 127.24, 127.04, 51.82, 21.09. NMR spectroscopic data agreed with literature values.[2]

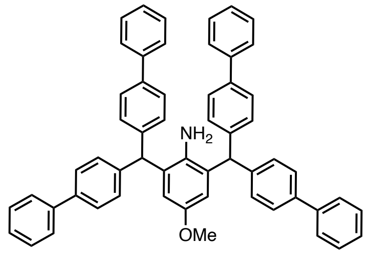

2,6-bis(Di([1,1'-biphenyl]-4-yl) methyl)-4-methoxylaniline (4)

An oven-dried 250 mL round-bottomed flask equipped with a stir bar was charged with mixture of p-Anisidine (1.66 g, 13.5 mmol, 1.0 equiv), di([1,1'-biphenyl]-4-yl) methanol (9.08 g, 27.0 mmol, 2.0 equiv) and anhydrous zinc chloride (0.92 g, 6.8 mmol, 0.5 equiv) in concentrated hydrochloric acid (1.13 mL, 37% in H_2O, 1.0 equiv). The mixture was heated to 160 °C and melted together. After 30 min at 160 °C, the reaction mixture started solidifying. The reaction mixture was cooled down to room temperature and dissolved in CH_2Cl_2 (100 mL). The CH_2Cl_2 layer was washed with water (3×100 mL) and NH_4Cl aqueous and dried over anhydrous Na_2SO_4. The solution was concentrated to 20 mL. The product was crashed out with 200 ml methanol and washed with methanol (3 × 100 mL). The desired aniline was obtained as white crystalline solid at 75.0 % (7.55 g) yield. ^{1}H NMR (500 MHz, $CDCl_3$) δ 7.62 (dd, J = 17.1, 7.7 Hz, 16H), 7.47 (t, J = 7.7 Hz, 8H), 7.38 (t, J = 7.6 Hz, 4H), 7.29 (d, J = 7.2 Hz, 8H), 6.41 (d, J = 3.2 Hz, 2H), 5.65 (s, 2H), 3.53 (d, J = 2.8 Hz, 3H), 3.37 (s, 2H). ^{13}C NMR (125 MHz, $CDCl_3$) δ 152.06, 141.57, 140.76, 139.65, 136.01, 130.93, 129.97, 128.81, 127.32 (d, J = 8.3 Hz), 114.50, 51.96. HRMS calcd for C57H45NO (M+ Na^+) 782.3398, found 782.3368. Elemental analysis of compound 4 has not been performed. This is not an organometallic compound. In this case, NMR and HRMS data were used to confirm the structure.

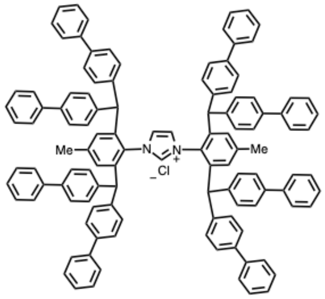

IPr$^{(4\text{-}Bp)}$•HCl (5)**

The mixture of 2,6-bis(di([1,1'-biphenyl]-4-yl) methyl)-4-methylaniline (1.5g, 2.0 mmol, 2.0 equiv), 40% glyoxal in water (160 mg, 1.1 mmol, 1.1 equiv), and paraformaldehyde (30 mg, 1.0 mmol, 1.0 equiv) in $CHCl_3$ (20 mL) was heated at 60 °C, and conc. HCl aq. (0.2 mL, 2.2 mmol, 1.1 equiv) was added. The mixture was stirred for 6 h at 60 °C before concentration in vacuo to give an off-white solid. The crude product was washed with Et_2O (3 × 25 mL) at room temperature and give IPr**$^{(4\text{-}Bp)}$•HCl with 65% yield (1.0 g) as white powder. ^{1}H NMR (500 MHz, $CDCl_3$) δ 13.27 (s, 1H), 7.55 (t, *J* = 5.8 Hz, 16H), 7.50 (d, *J* = 7.4 Hz, 8H), 7.45 – 7.30 (m, 40H), 6.95 (d, *J* = 7.2 Hz, 12H), 5.80 (s, 2H), 5.45 (s, 4H), 2.29 (s, 6H).^{13}C NMR (125 MHz, $CDCl_3$) δ 141.90, 141.48, 140.65, 130.92, 130.50, 129.72, 128.91, 128.73, 127.60, 127.35, 127.17, 127.03, 126.79, 65.88, 50.82, 21.94, 15.31. HRMS calcd for $C_{117}H_{89}N_2$ (M+H$^+$)1522.7104, found 1522.7174. Elemental analysis of compound **5** has not been performed. This is not an organometallic compound. In this case, NMR and HRMS data were used to confirm the structure.

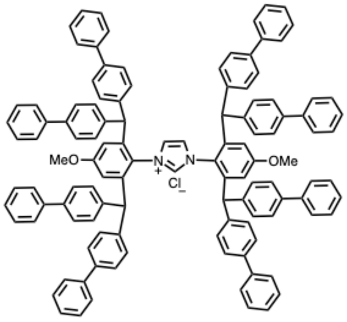

IPr$^{MeO(4\text{-}Bp)}$•HCl (6)**

The mixture of 2,6-bis(di([1,1'-biphenyl]-4-yl) methyl)-4-methoxylaniline (1.5g, 2.0 mmol, 2.0 equiv), 40% glyoxal in water (160 mg, 1.1 mmol, 1.1 equiv), and paraformaldehyde (30 mg, 1.0 mmol, 1.0 equiv) in $CHCl_3$ (20 mL) was heated at 60 °C, and conc. HCl aq. (0.2 mL, 2 mmol, 2

equiv) was added. The mixture was stirred for 6 h at 60 °C before concentration in vacuo to give an off-white solid. The crude product was washed with Et_2O (3 × 25 mL) at room temperature and give IPr**$^{Meo(4-Bp)}$•HCl 72% yield (1.15g) as white powder. ^{1}H NMR (500 MHz, $CDCl_3$): δ 13.17 (s, 1H), 7.55 (d, *J* = 7.5 Hz, 16H), 7.49 (d, *J* = 7.4 Hz, 8H), 7.46 – 7.30 (m, 40H), 6.98 (d, *J* = 7.7 Hz, 8H), 6.65 (s, 4H), 5.73 (s, 2H), 5.47 (s, 4H), 3.60 (s, 6H). ^{13}C NMR (125 MHz, $CDCl_3$) δ 142.74, 141.33, 140.79, 140.57, 139.80, 139.70, 139.58, 130.48, 129.66, 128.90, 128.73, 127.19, 127.02, 126.79, 115.71, 55.37, 51.03. HRMS calcd for $C_{117}H_{89}N_2O_2$(M+H$^+$)1554.7002, found 1554.7068. Elemental analysis of compound **6** has not been performed. This is not an organometallic compound. In this case, NMR and HRMS data were used to confirm the structure.

General Procedure for the Synthesis of IPr$^{(4-Bp)}$ Complexes**

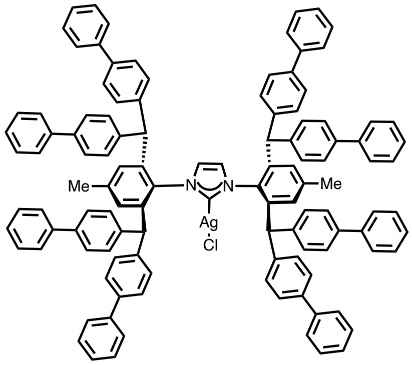

[Ag(IPr$^{(4-Bp)}$)Cl] (7)**

An oven-dried flask equipped with a stir bar was charged with IPr**$^{(4-Bp)}$•HCl (312 mg, 0.2 mmol, 1.1 equiv) and silver oxide (42 mg, 0.18 mmol, 1 equiv), placed under a positive pressure of argon and subjected to three evacuation/backfilling cycles under high vacuum. CH_2Cl_2 (4 mL) was added, and the resulting reaction mixture was stirred at room temperature. The resulting suspension was stirred away from light overnight. The reaction mixture was filtered through Celite with dichloromethane as eluent, concentrated under reduced pressure, and dried under high vacuum to afford 285 mg of [Ag(IPr**$^{(4-Bp)}$)Cl] as white solid (95% yield). ^{1}H NMR (500 MHz, $CDCl_3$) δ 7.48 (d, *J* = 7.6 Hz, 8H), 7.42 (d, *J* = 7.8 Hz, 8H), 7.38 (d, *J* = 7.4 Hz, 8H), 7.34 (t, *J* = 7.5 Hz, 8H), 7.30 – 7.18 (m, 24H), 7.00 (d, *J* = 7.8 Hz, 8H), 6.90 (d, *J* = 7.8 Hz, 8H), 6.86 (s, 4H), 6.14 (s, 2H), 5.13 (s, 4H), 2.19 (s, 6H). ^{13}C NMR (151 MHz, $CDCl_3$) δ 142.20, 140.95, 140.88, 140.61, 140.28, 139.67, 139.23, 134.49, 130.40, 129.95, 129.70, 128.88, 128.67, 127.59, 126.92, 50.59, 21.85. HRMS calcd for $C_{117}H_{88}N_2ClAg$ (M + H$^+$)1663.5765, found 1663.5758.

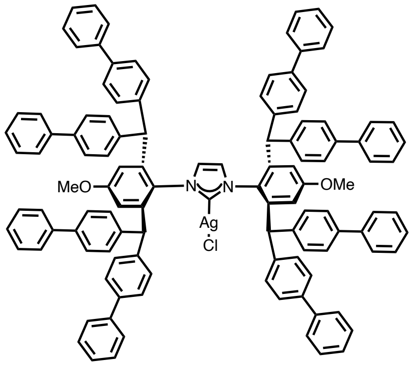

[Ag(IPr^MeO(4-Bp)^)Cl] (8)**

An oven-dried flask equipped with a stir bar was charged with IPr**^Meo(4-Bp)^•HCl (365 mg, 0.23 mmol, 1.1 equiv) and silver oxide (49 mg, 0.21 mmol, 1 equiv), placed under a positive pressure of argon and subjected to three evacuation/backfilling cycles under high vacuum. CH_2Cl_2 (4 mL) was added, and the resulting reaction mixture was stirred at room temperature. The resulting suspension was stirred away from light overnight. The reaction mixture was filtered through Celite with dichloromethane as eluent, concentrated under reduced pressure, and dried under high vacuum to afford 313 mg of [Ag(IPr**^MeO(4-Bp)^)Cl] as white solid (88% yield). ^{1}H NMR (600 MHz, $CDCl_3$) 7.48 (d, J = 7.6 Hz, 8H), 7.44 – 7.40 (m, 8H), 7.39 (d, J = 7.4 Hz, 8H), 7.34 (t, J = 7.3 Hz, 8H), 7.27 (q, J = 6.6 Hz, 12H), 7.23 – 7.19 (m, 12H), 7.01 (d, J = 7.7 Hz, 8H), 6.92 (d, J = 7.8 Hz, 8H), 6.57 (d, J = 2.1 Hz, 4H), 6.11 (s, 2H), 5.13 (s, 4H), 3.53 (s, 6H). ^{13}C NMR (125 MHz, $CDCl_3$) δ 160.29, 142.77, 142.01, 140.71, 140.58, 140.24, 139.77, 139.36, 129.90, 129.68, 128.89, 127.66, 127.19, 126.92, 115.15, 55.33, 50.84. HRMS calcd for $C_{117}H_{88}N_2AgO_2$ (M) 1659.5896, found 1659.5905. Anal. Calcd for $C_{117}H_{88}N_2AgO_2Cl$: C, 82.79; H, 5.23; N, 1.65. Found: C, 82.63; H, 5.07; N, 1.63.

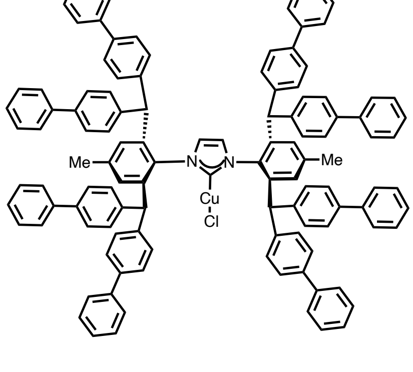

[Cu(IPr$^{(4\text{-}Bp)}$)Cl] (9)**

An oven-dried flask equipped with a stir bar was charged with copper chloride (40 mg, 0.4 mmol, 2 equiv), IPr**$^{(4\text{-}Bp)}$•HCl (312 mg, 0.2 mmol, 1 equiv) and KO*t*Bu (28 mg, 0.24 mmol, 1.2 equiv), placed under a positive pressure of argon and subjected to three evacuation/backfilling cycles under high vacuum. THF (4mL) was added, and the resulting reaction mixture was stirred at room temperature for 12 h. The reaction mixture was filtered through Celite with dichloromethane as eluent, concentrated under reduced pressure, and dried under high vacuum to afford 178 mg of [Cu(IPr**$^{(4\text{-}Bp)}$)Cl] as pale yellow solid (55% yield). ^{1}H NMR (500 MHz, $CDCl_3$) δ 7.49 – 7.43 (m, 8H), 7.38 (td, *J* = 8.1, 1.4 Hz, 8H), 7.32 (dd, *J* = 8.3, 6.7 Hz, 16H), 7.28 – 7.19 (m, 24H), 7.02 (d, *J* = 8.1 Hz, 8H), 6.98 (d, *J* = 8.3 Hz, 8H), 6.89 (s, 4H), 6.02 (s, 2H), 5.22 (s, 4H), 2.19 (s, 6H). ^{13}C NMR (125 MHz, $CDCl_3$) δ 180.39, 142.14, 141.27, 140.93, 140.68, 140.28, 139.55, 139.20, 134.35, 130.31, 129.92, 128.86, 127.44, 127.09, 126.90, 50.67, 21.89. HRMS calcd for $C_{117}H_{88}N_2CuCl$ (M-CuCl) 1520.6947, found 1520.7004. Anal. Calcd for $C_{117}H_{88}N_2CuCl+H_2O$: C, 85.74; H, 5.54; N, 1.71. Found: C, 85.58; H, 5.72; N, 1.58.

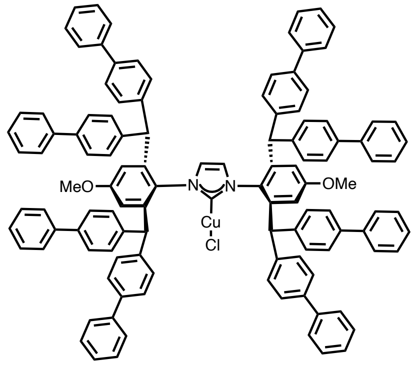

[Cu(IPr$^{MeO(4\text{-}Bp)}$)Cl] (10)**

An oven-dried flask equipped with a stir bar was charged with copper chloride (40 mg, 0.4 mmol, 2 equiv), IPr**$^{MeO(4\text{-}Bp)}$•HCl (317 mg, 0.2 mmol, 1 equiv) and KO*t*Bu (28 mg, 0.24 mmol, 1.2 equiv), placed under a positive pressure of argon and subjected to three evacuation/backfilling cycles under high vacuum. THF (4mL) was added, and the resulting reaction mixture was stirred at room temperature for 12 h. The reaction mixture was filtered through Celite with dichloromethane as eluent, concentrated under reduced pressure, and dried under high vacuum to afford 158 mg of [Cu(IPr**$^{MeO(4\text{-}Bp)}$)Cl] as pale yellow solid (48% yield). ^{1}H NMR (500 MHz, $CDCl_3$) δ 7.49 – 7.43 (m, 8H), 7.42 – 7.36 (m, 16H), 7.32 (t, *J* = 7.6 Hz, 8H), 7.25 (dd, *J* = 8.5, 6.3 Hz, 20H), 7.23 – 7.19 (m, 4H), 7.04 (d, *J* = 8.1 Hz, 8H), 6.99 (d, *J* = 8.2 Hz, 8H), 6.59 (s, 4H),

5.98 (s, 2H), 5.22 (s, 4H), 3.53 (s, 6H). ^{13}C NMR (125 MHz, $CDCl_3$) δ 160.25, 142.82, 141.93, 141.03, 140.65, 140.24, 139.65, 139.33, 129.85, 128.87, 128.67, 127.51, 127.10, 126.90, 115.04, 55.30, 50.91. Anal. Calcd for $C_{117}H_{88}N_2O_2ClCu+H_2O$: C, 84.10; H, 5.43; N, 1.69. Found: C, 84.50; H, 5.82; N, 1.70.

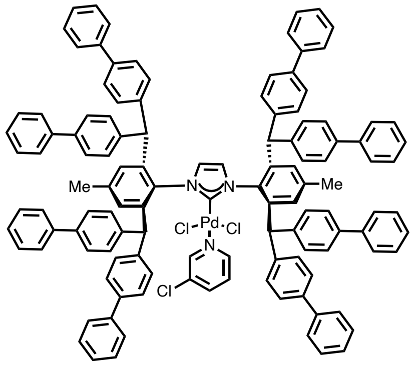

[Pd(IPr$^{(4-Bp)}$)(3-Cl-py)Cl_2] (11)**

An oven-dried flask equipped with a stir bar was charged with $PdCl_2$ (40 mg, 0.23 mmol, 1 equiv), IPr**$^{MeO(4-Bp)}$•HCl (390 mg, 0.25 mmol, 1.1 equiv), K_2CO_3 (160 mg, 1.15 mmol, 5 equiv) and 3-chloropyridine (4.0 mL). The resulting reaction mixture was placed in a preheated oil bath at 80 °C and stirred for 16 h at 80 °C. After the indicated time, the reaction mixture was cooled to room temperature, diluted with CH_2Cl_2 (10 mL), passed through a short pad of silica eluting with CH_2Cl_2, and concentrated. The pure [Pd(IPr**$^{(4-Bp)}$)(3-Cl-py)Cl_2] (355 mg, 85%) was isolated by trituration with hexane, decanting the supernatant, washing the solid with hexane and drying under high vacuum. ^1H NMR (500 MHz, $CDCl_3$) δ 9.32 (s, 1H), 9.15 (d, J = 5.5 Hz, 1H), 7.87 (d, J = 8.2 Hz, 1H), 7.63 (d, J = 7.7 Hz, 8H), 7.59 (d, J = 8.1 Hz, 8H), 7.54 (d, J = 8.1 Hz, 8H), δ 7.44 (t, J = 7.6 Hz, 9H), 7.38 (d, J = 7.2 Hz, 8H), 7.34 – 7.30 (m, 16H).7.22 (d, J = 7.8 Hz, 8H), 6.94 (s, 4H), 6.87 (d, J = 7.8 Hz, 8H), 6.49 (s, 4H), 5.07 (s, 2H), 2.29 (s, 6H). ^{13}C NMR (125 MHz, $CDCl_3$) δ 150.95, 149.87, 143.50, 142.95, 141.70, 140.27, 138.87, 138.80, 135.37, 131.02, 130.86, 129.92, 128.72, 127.18, 127.05, 127.01, 126.78, 126.61, 124.82, 124.46, 50.39, 21.89. Anal. Calcd for $C_{122}H_{92}N_2Cl_3Pd+CH_2Cl_2$: C, 78.42; H, 5.03; N, 1.48. Found: C, 78.01; H, 4.91; N, 1.34.

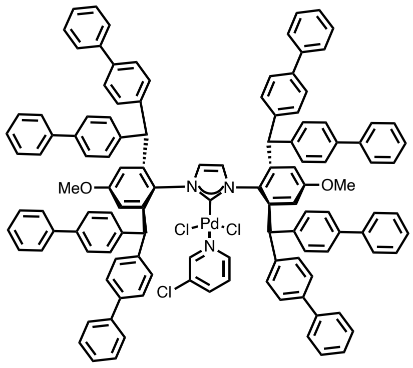

[Pd(IPr$^{MeO(4-Bp)}$)(3-Cl-py)Cl_2] (12)**

An oven-dried flask equipped with a stir bar was charged with $PdCl_2$ (40 mg, 0.23 mmol, 1 equiv), IPr**OMe•HCl (400 mg, 0.25 mmol, 1.1 equiv), K_2CO_3 (160 mg, 1.15 mmol, 5 equiv) and 3-chloropyridine (4.0 mL). The resulting reaction mixture was placed in a preheated oil bath at 80 °C and stirred for 16 h at 80 °C. After the indicated time, the reaction mixture was cooled to room temperature, diluted with CH_2Cl_2 (10 mL), passed through a short pad of silica eluting with CH_2Cl_2, and concentrated. The pure [Pd(IPr**$^{MeO(4-Bp)}$)(3-Cl-py)Cl_2] (290 mg, 70%) was isolated by trituration with hexane, decanting the supernatant, washing the solid with hexane and drying under high vacuum. ^{1}H NMR (500 MHz, $CDCl_3$) δ 9.32 (s, 1H), 9.15 (d, *J* = 5.5 Hz, 1H), 7.88 (d, *J* = 8.3 Hz, 1H), 7.64 – 7.59 (m, 16H), 7.54 (d, *J* = 8.1 Hz, 8H), 7.44 (t, *J* = 7.6 Hz, 12H), 7.38 (s, 9H), 7.34 – 7.30 (m, 12H), 7.23 (d, *J* = 7.8 Hz, 8H), 6.90 (d, *J* = 7.9 Hz, 8H), 6.67 (s, 4H), 6.47 (s, 4H), 4.99 (s, 2H), 3.61 (s, 6H). ^{13}C NMR (125 MHz, $CDCl_3$) δ 159.12, 150.88, 149.79, 143.78, 143.16, 142.82, 140.96, 140.23, 139.05, 138.90, 130.86, 129.85, 128.76, 128.72, 127.21, 127.02, 126.86, 126.79, 126.70, 115.54, 55.05, 50.63. Anal. Calcd for $C_{122}H_{92}N_2Cl_3O_2Pd$+$CH_2Cl_2$: C, 77.11; H, 4.95; N, 1.46. Found: C, 77.23; H, 4.86; N, 1.27.

ORTEP Structures of 7 and 9 and 11 (Figure S1-S3)

Figure S1. *ORTEP Structure of* **7** *(50% ellipsoids).* (Crystallographic data has been deposited with the Cambridge Crystallographic Data Center as supplementary publication No. 2429423).

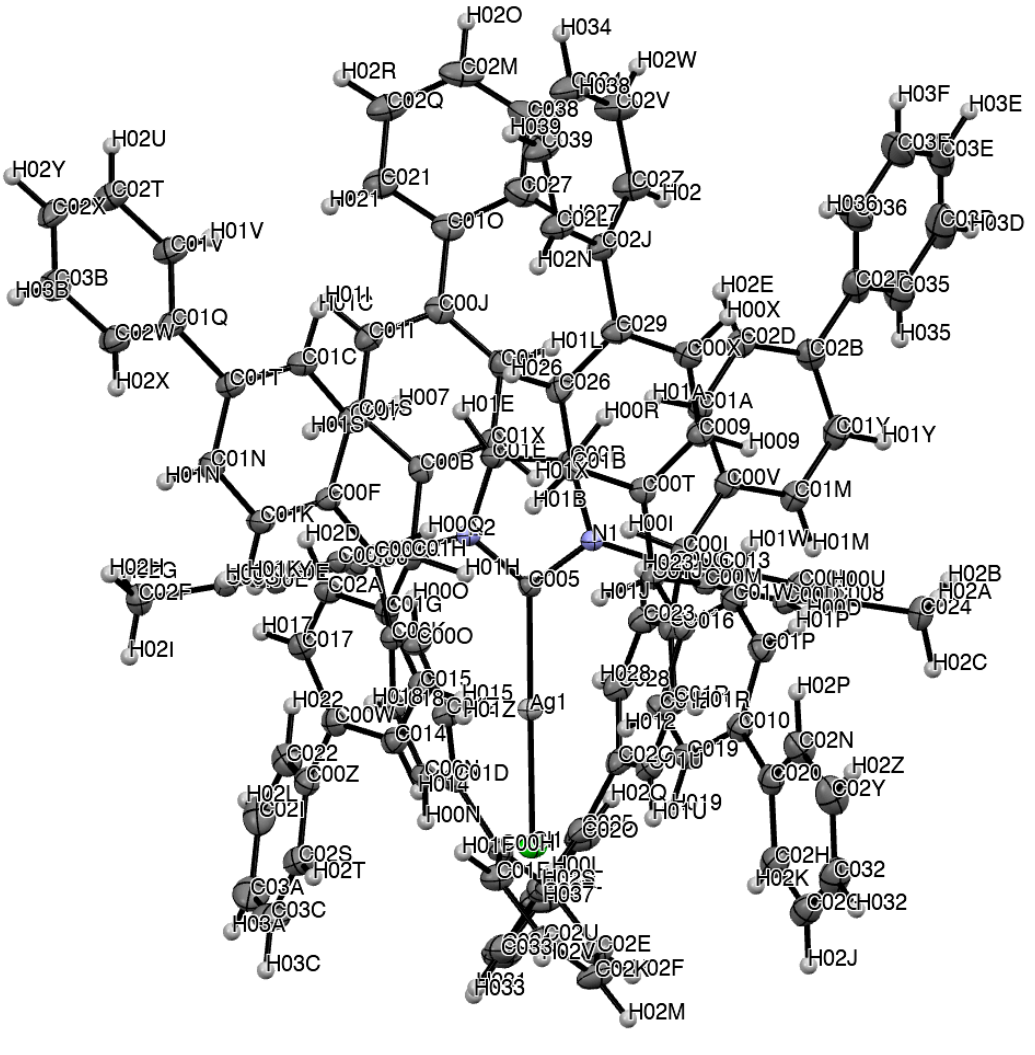

Selected bond lengths [Å] and angles [°]: Ag1–Cl1, 2.3369; Ag–C005, 2.091; C005–N2, 1.350; C005–N1, 1.349; N1–C00R, 1.390; N2–C01E, 1.393; N2–C00A, 1.436; N1–C00G, 1.443; C005–Ag–Cl1, 179.16; Ag1–C005–N2, 129.15; Ag1–C005–N1, 126.29; N1–C005–N2, 104.53; N1–C00R–C01E, 107.01; N2–C01E–C00R, 106.24; C005–N2–C00A, 126.23; C005–N1–C00G, 126.03.

Figure S2. *ORTEP Structure of* **9** *(50% ellipsoids).* (Crystallographic data has been deposited with the Cambridge Crystallographic Data Center as supplementary publication No. 2429653).

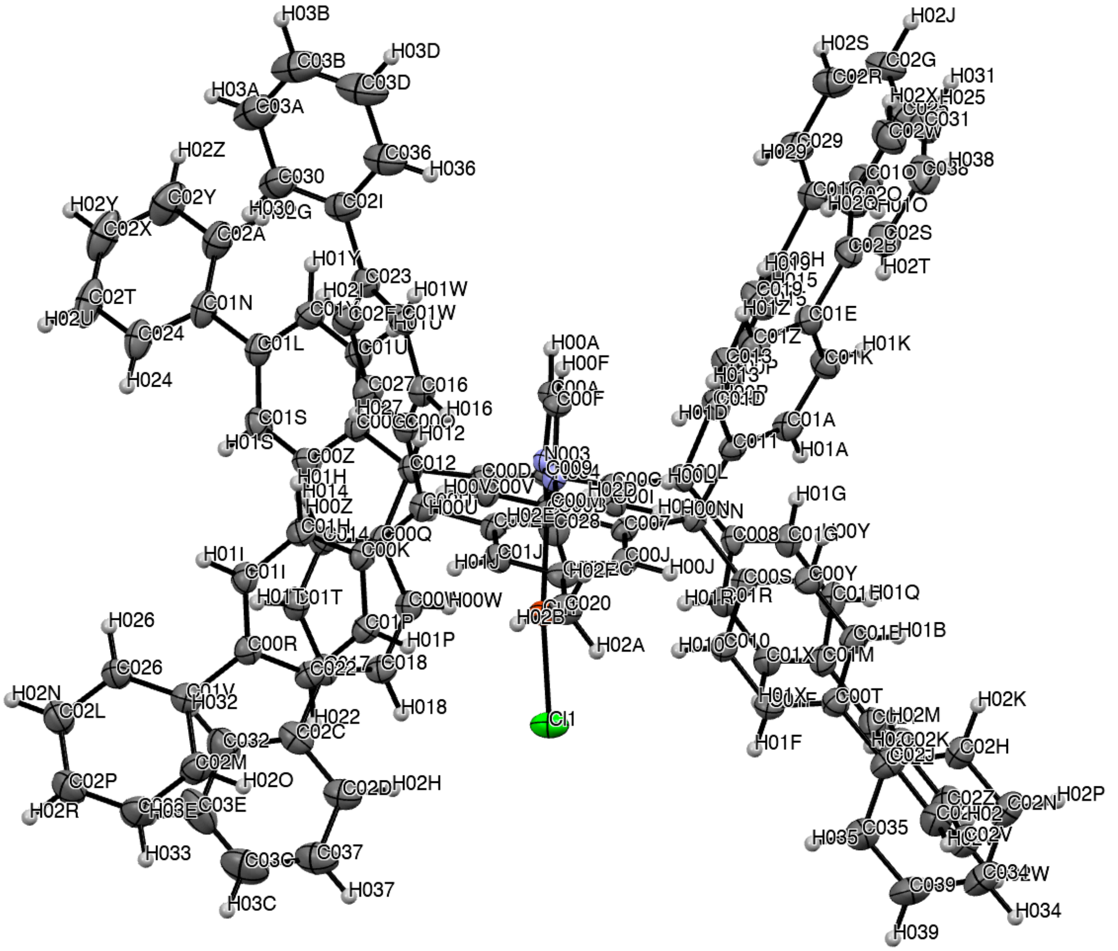

Selected bond lengths [Å] and angles [°]: Cu1–Cl1, 2.107; Cu–C006, 1.880; C006–N004, 1.361; C006–N003, 1.380; N003–C00A, 1.387; N004–C00F, 1.381; N003–C009, 1.442; N004–C00B, 1.446; Cl1–Cu1–C006, 174.09; Cu1–C006–N004, 127.50; Cu1–C006–N003, 128.82; N003–C006–N004, 103.67; C006–N004–C00F, 111.44; C006–N004–C00B, 123.86; C006–N003–C009, 123.07.

Figure S3. *ORTEP Structure of* **11** *(50% ellipsoids).* (Crystallographic data has been deposited with the Cambridge Crystallographic Data Center as supplementary publication No. 2429424).

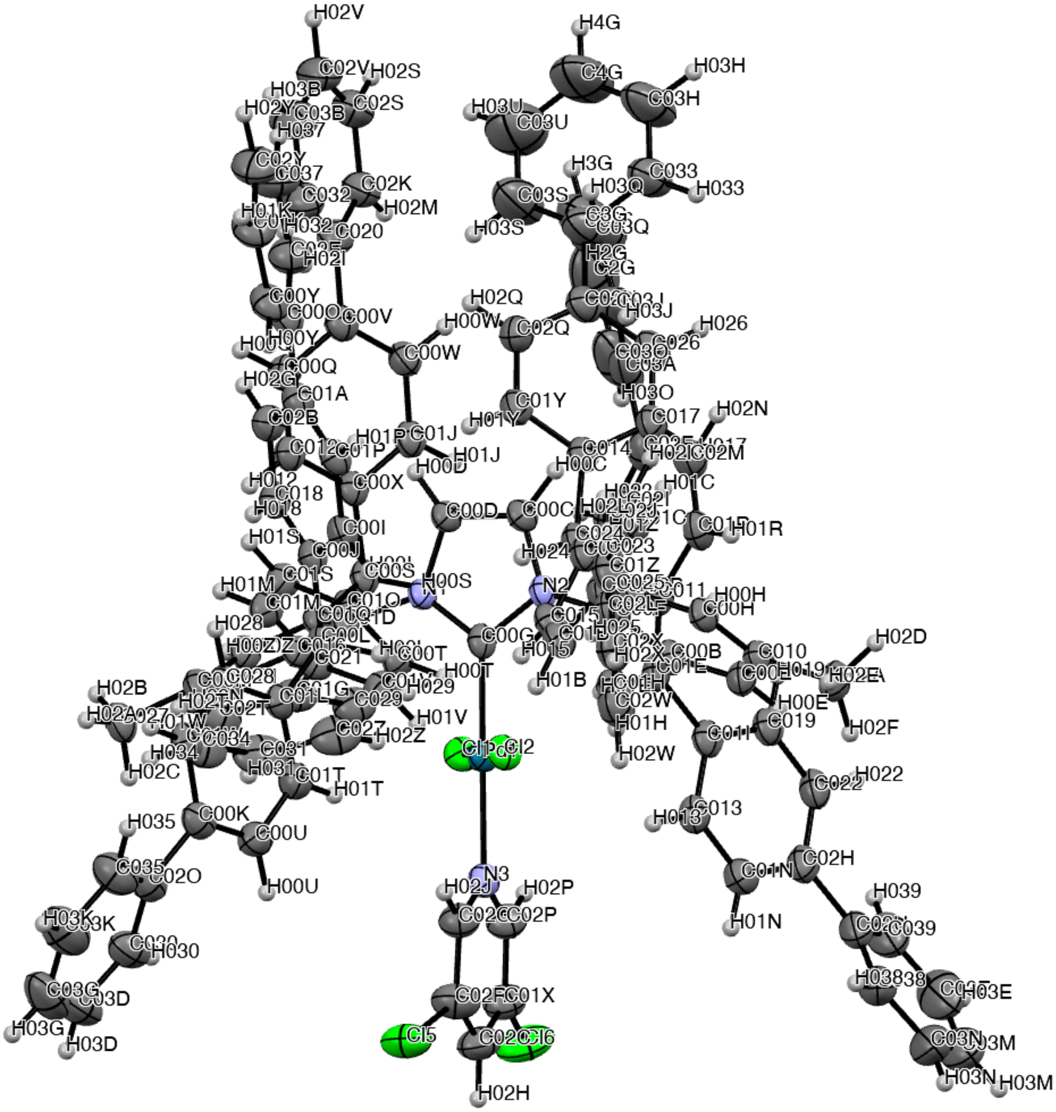

Selected bond lengths [Å] and angles [°]: Pd1–C00G, 1.965; Pd1–N3, 2.119; N2–C00G, 1.362; N1–C00G, 1.359; N2–C00C, 1.383; N1–C00D, 1.393; C00D–C00C, 1.341; N1–C01D, 1.448; N2–C00F, 1.448; N3–C02G, 1.333; N3–C02P, 1.348; Pd1–N3–C02P, 121.7; Pd1–N3–C02G, 119.89; N3–Pd1–C00G, 178.01; Cl2–Pd1–C00G, 89.80; Cl1–Pd1–C00G, 87.57; Pd1–C00G–N1, 126.96; Pd1–C00G–N2, 127.68; C00G–N2–C00C, 110.44; C00G–N1–C00D, 110.30; C00G–N1–C01D, 123.5; C00G–N2–C00F, 124.5.

Table S1. *Crystal Data and Structure Refinement Summaries for* **7**.

Compound	**7**
Chemical formula	$C_{117}H_{88}AgClN_2 \cdot 0.5(C_4H_6O_2) \cdot 2(C_3H_6O)$
M_r	1824.41
Crystal system, space group	Monoclinic, $P2_1/n$
Temperature (K)	100
a, *b*, *c* (Å)	16.6367 (1), 19.7184 (1), 30.6391 (2)
β (°)	101.690 (1)
α, β, γ (°)	78.929 (1), 81.664 (2), 76.007 (1)
V (Å^3)	9842.65 (11)
Z	4
Radiation type	Cu *K*α
μ (mm^{-1})	2.32
Crystal size (mm)	0.17 × 0.06 × 0.04
Diffractometer	XtaLAB Synergy, Dualflex, HyPix
Absorption correction	Multi-scan<br>*CrysAlis PRO* 1.171.43.100a (Rigaku Oxford Diffraction, 2023) Empirical absorption correction using spherical harmonics, implemented in SCALE3 ABSPACK scaling algorithm.
T_{min}, T_{max}	0.817, 1.000
No. of measured, independent and observed [$I > 2\sigma(I)$] reflections	172764, 19888, 16078
R_{int}	0.094
(sin θ/λ)$_{max}$ (Å^{-1})	0.631
$R[F^2 > 2\sigma(F^2)]$, $wR(F^2)$, S	0.040, 0.108, 1.01
No. of reflections	19888
No. of parameters	1196
H-atom treatment	H-atom parameters constrained
$\Delta\rho_{max}$, $\Delta\rho_{min}$ (e Å^{-3})	0.61, −0.74

Computer programs: *CrysAlis PRO* 1.171.42.58a (Rigaku OD, 2022), *SHELXL2018*/3 (Sheldrick, 2018), *SHELXTL*.

Table S2. *Crystal Data and Structure Refinement Summaries for* **9**.

Compound	**9**
Chemical formula	$C_{117}H_{88}ClCuN_2$
M_r	1620.88
Crystal system, space group	Monoclinic, $P2_1/n$
Temperature (K)	102
a, *b*, *c* (Å)	16.5831 (1), 30.0372 (3), 20.5259 (2)
β (°)	104.207 (1)
V (Å^3)	9911.45 (16)
Z	4
Radiation type	Cu *K*α
μ (mm^{-1})	0.91
Crystal size (mm)	0.18 × 0.16 × 0.08
Diffractometer	Bruker *SMART* CCD Apex-II area-detector
Absorption correction	Multi-scan *CrysAlis PRO* 1.171.43.100a (Rigaku Oxford Diffraction, 2023) Empirical absorption correction using spherical harmonics, implemented in SCALE3 ABSPACK scaling algorithm.
T_{min}, T_{max}	0.877, 1.000
No. of measured, independent and observed [$I > 2\sigma(I)$] reflections	132125, 19395, 14740
R_{int}	0.100
$(\sin \theta/\lambda)_{max}$ (Å^{-1})	0.631
$R[F^2 > 2\sigma(F^2)]$, $wR(F^2)$, S	0.048, 0.134, 1.04
No. of reflections	19395
No. of parameters	1092
H-atom treatment	H-atom parameters constrained
$\Delta\rho_{max}$, $\Delta\rho_{min}$ (e Å^{-3})	0.41, −0.49

Computer programs: *CrysAlis PRO* 1.171.42.58a (Rigaku OD, 2022), *SHELXL2018*/3 (Sheldrick, 2018), *SHELXTL*.

Table S3. *Crystal Data and Structure Refinement Summaries for* **11**.

Compound	**11**
Chemical formula	$C_{122}H_{91}Cl_3N_3Pd{\cdot}CH_2Cl_2$
M_r	1896.65
Crystal system, space group	Triclinic, $P\bar{1}$
Temperature (K)	100
a, *b*, *c* (Å)	13.7414 (1), 17.3871 (2), 23.7335 (3)
α, β, γ (°)	84.474 (1), 83.221 (1), 84.831 (1)
V (Å^3)	5586.86 (11)
Z	2
Radiation type	Cu *K*α
μ (mm^{-1})	2.79
Crystal size (mm)	0.23 × 0.20 × 0.11
Diffractometer	XtaLAB Synergy, Dualflex, HyPix
Absorption correction	Multi-scan<br>*CrysAlis PRO* 1.171.43.143a (Rigaku Oxford Diffraction, 2024) Empirical absorption correction using spherical harmonics, implemented in SCALE3 ABSPACK scaling algorithm.
T_{min}, T_{max}	0.764, 1.000
No. of measured, independent and observed [$I > 2\sigma(I)$] reflections	117888, 21504, 18948
R_{int}	0.056
(sin θ/λ)$_{max}$ (Å^{-1})	0.629
$R[F^2 > 2\sigma(F^2)]$, $wR(F^2)$, S	0.051, 0.149, 1.05
No. of reflections	21504
No. of parameters	1200
No. of restraints	48
H-atom treatment	H-atom parameters constrained
$\Delta\rho_{max}$, $\Delta\rho_{min}$ (e Å^{-3})	1.62, −0.94

Computer programs: *CrysAlis PRO* 1.171.42.58a (Rigaku OD, 2022), *SHELXL2018*/3 (Sheldrick, 2018), *SHELXTL*.

Computational Methods

Computational Methods. All the calculations were performed using Gaussian 09 suite of programs. All of the geometry optimizations were performed at the B3LYP level of theory in the gas phase with the 6-311++G(d,p) basis set. For geometry optimizations, we employed the X-ray structures of [(IPr**($^{4-Bp)}$)AgCl] and [(IPr**($^{4-Bp)}$)CuCl] as the starting geometry and performed full optimization. The structures of both the free carbene and the corresponding metal complexes have been fully reoptimized in vacuo. The absence of imaginary frequencies was used to characterize the structures as minima on the potential energy surface. All of the optimized geometries were verified as minima (no imaginary frequencies). Energetic parameters were calculated under standard conditions (298.15 K and 1 atm). Structural representations were generated using CYLview software (Legault, C. Y. CYL view version 1.0 BETA, University of Sherbrooke). All other representations were generated using Gauss View (GaussView, version 5, Dennington, R.; Keith, T.; Millam, J. Semichem Inc., Shawnee Mission, KS, 2009) or ChemCraft software (Andrienko, G. L. ChemCraft version b562a, https://www.chemcraftprog.com).

Full Reference for Gaussian 09

Gaussian 09, Revision D.01, Frisch, M. J.; Trucks, G. W.; Schlegel, H. B.; Scuseria, G. E.; Robb, M. A.; Cheeseman, J. R.; Scalmani, G.; Barone, V.; Mennucci, B.; Petersson, G. A.; Nakatsuji, H.; Caricato, M.; Li, X.; Hratchian, H. P.; Izmaylov, A. F.; Bloino, J.; Zheng, G.; Sonnenberg, J. L.; Hada, M.; Ehara, M.; Toyota, K.; Fukuda, R.; Hasegawa, J.; Ishida, M.; Nakajima, T.; Honda, Y.; Kitao, O.; Nakai, H.; Vreven, T.; Montgomery, J. A., Jr.; Peralta, J. E.; Ogliaro, F.; Bearpark, M.; Heyd, J. J.; Brothers, E.; Kudin, K. N.; Staroverov, V. N.; Kobayashi, R.; Normand, J.; Raghavachari, K.; Rendell, A.; Burant, J. C.; Iyengar, S. S.; Tomasi, J.; Cossi, M.; Rega, N.; Millam, M. J.; Klene, M.; Knox, J. E.; Cross, J. B.; Bakken, V.; Adamo, C.; Jaramillo, J.; Gomperts, R.; Stratmann, R. E.; Yazyev, O.; Austin, A. J.; Cammi, R.; Pomelli, C.; Ochterski, J. W.; Martin, R. L.; Morokuma, K.; Zakrzewski, V. G.; Voth, G. A.; Salvador, P.; Dannenberg, J. J.; Dapprich, S.; Daniels, A. D.; Farkas, Ö.; Foresman, J. B.; Ortiz, J. V.; Cioslowski, J.; Fox, D. J. Gaussian, Inc., Wallingford CT, 2009.

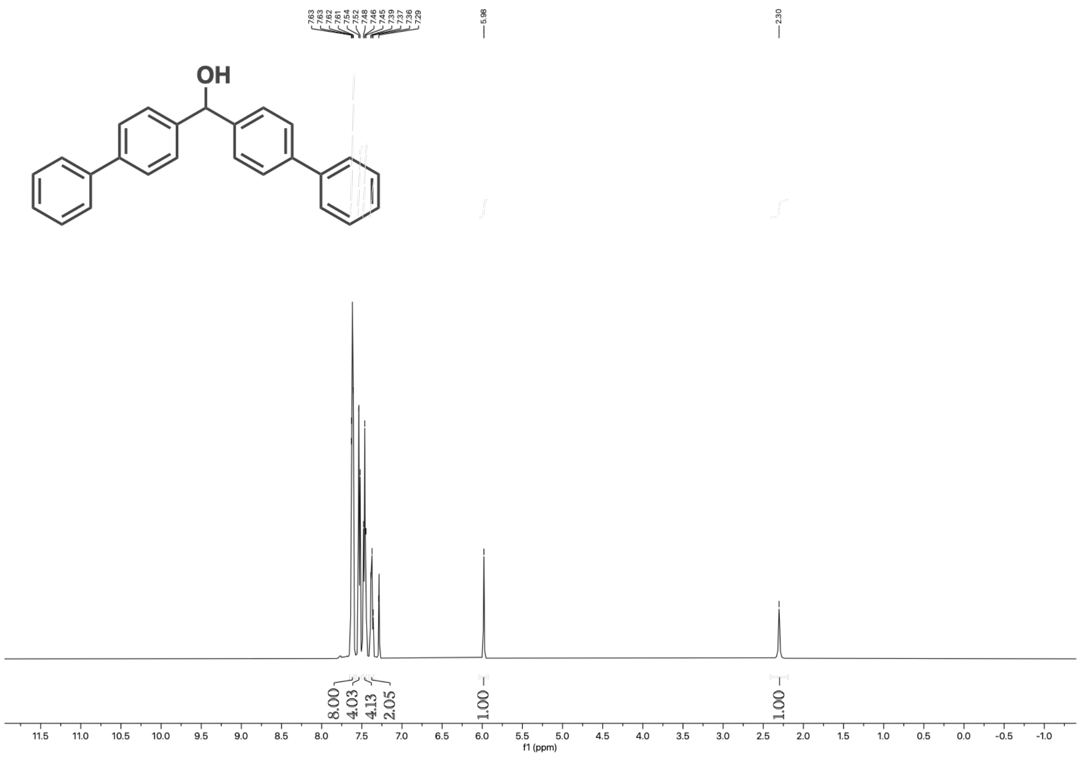

Figure S4 ^{1}H NMR (500 MHz, $CDCl_3$) Spectrum of **2**

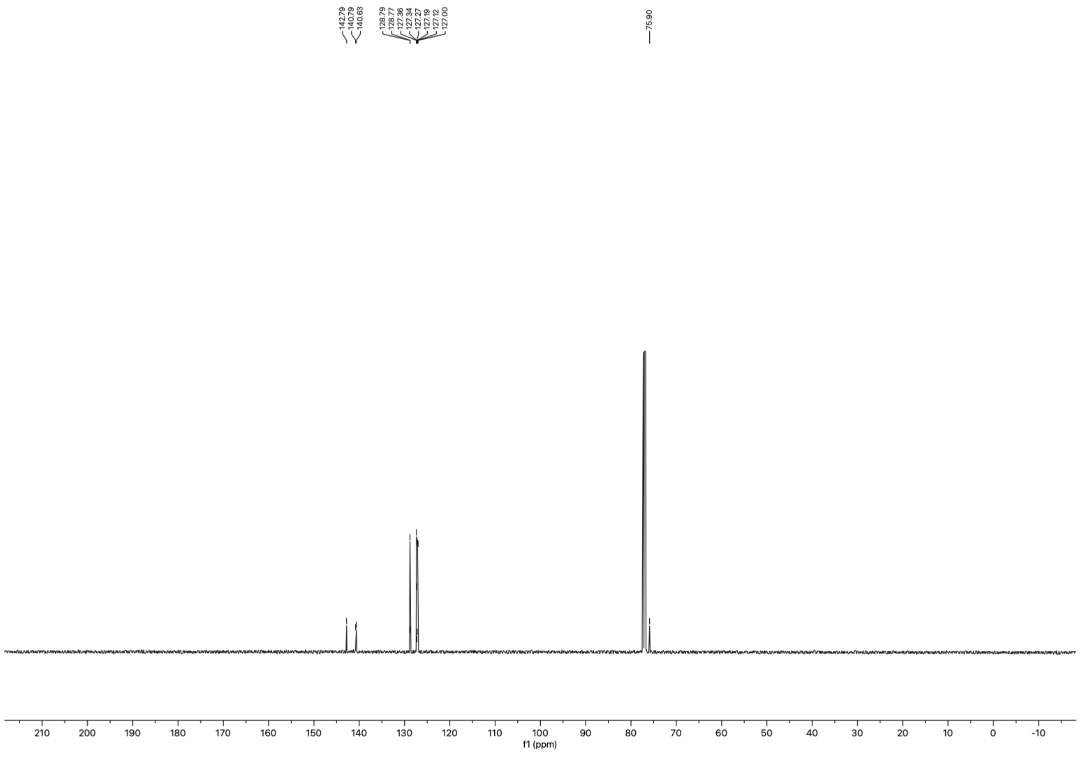

Figure S5 ^{13}C {^{1}H} NMR (125 MHz, $CDCl_3$) Spectrum of **2**

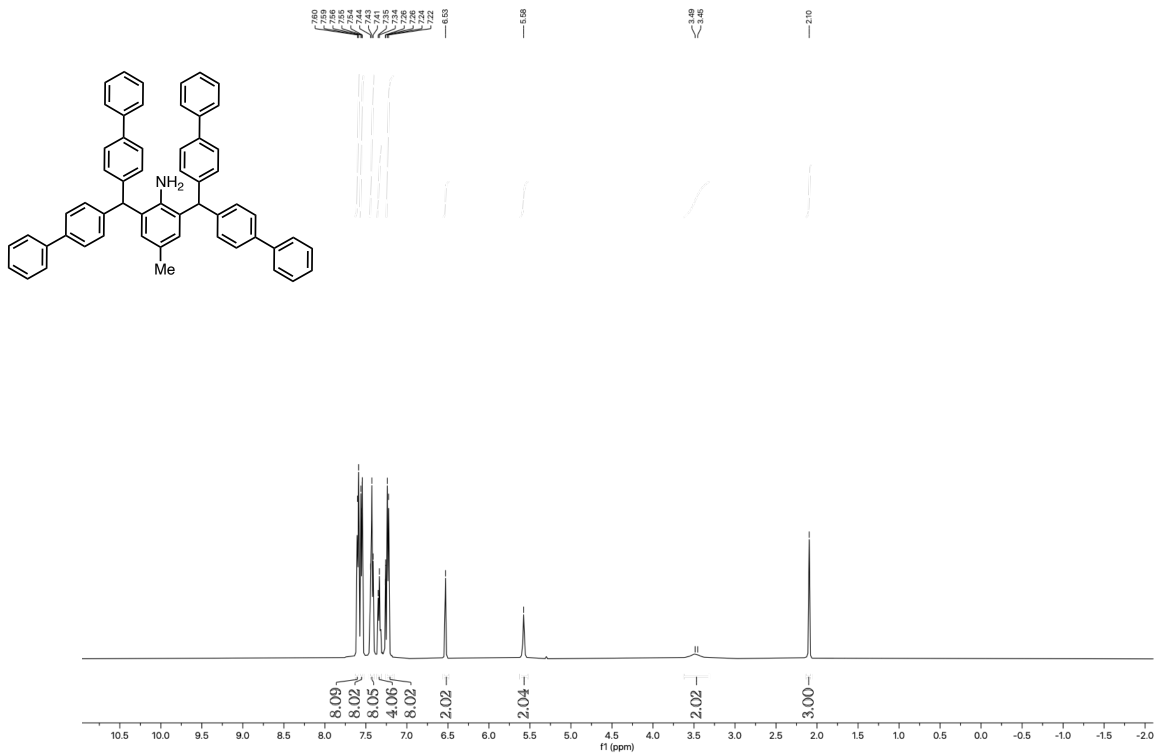

Figure S6 ^{1}H NMR (500 MHz, $CDCl_3$) Spectrum of **3**

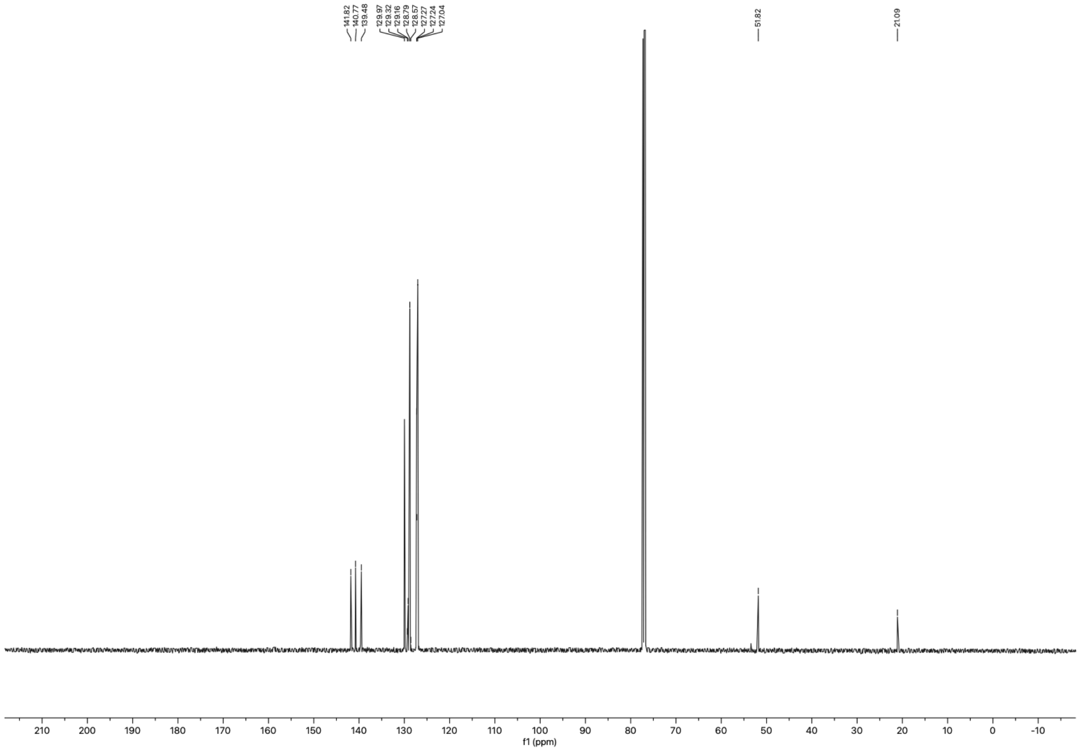

Figure S7 ^{13}C {^{1}H} NMR (125 MHz, $CDCl_3$) Spectrum of **3**

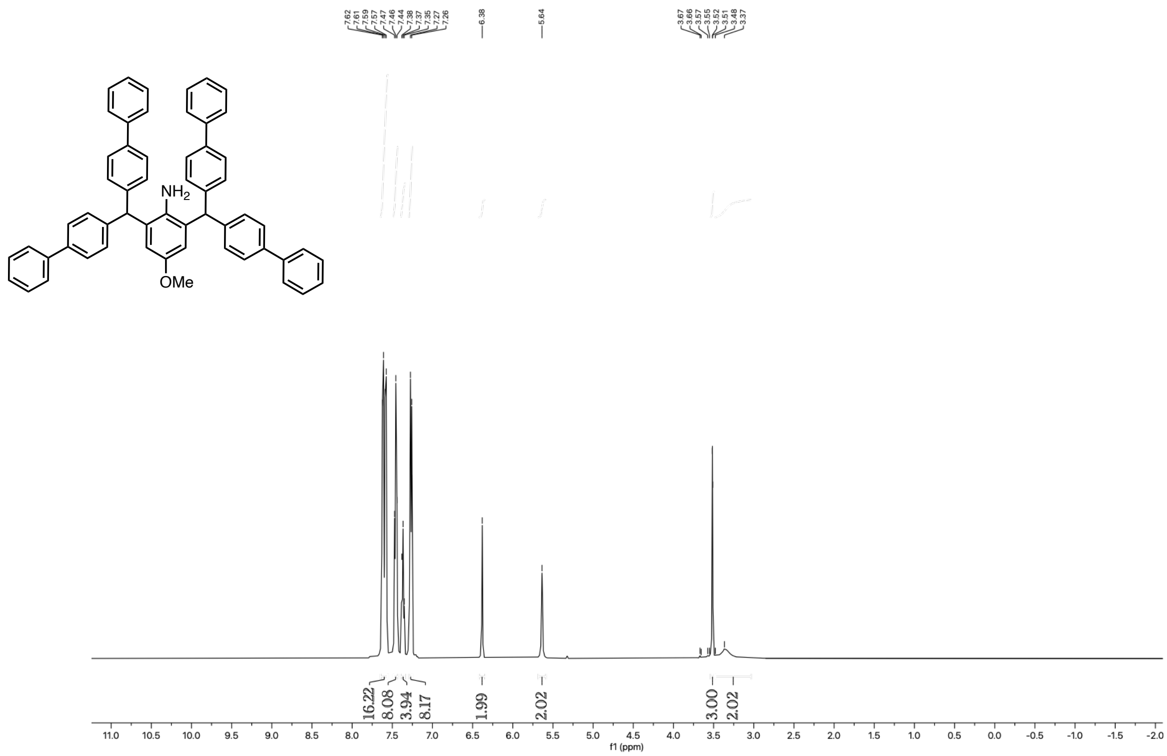

Figure S8 ^{1}H NMR (500 MHz, $CDCl_3$) Spectrum of **4**

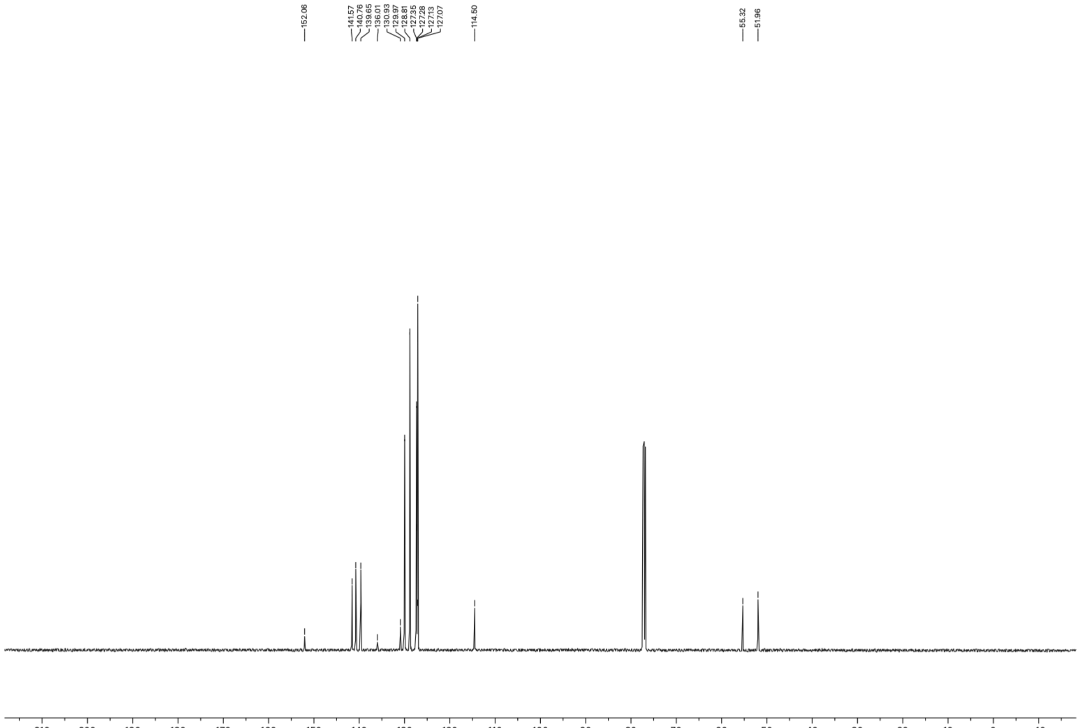

Figure S9 ^{13}C {^{1}H} NMR (125 MHz, $CDCl_3$) Spectrum of **4**

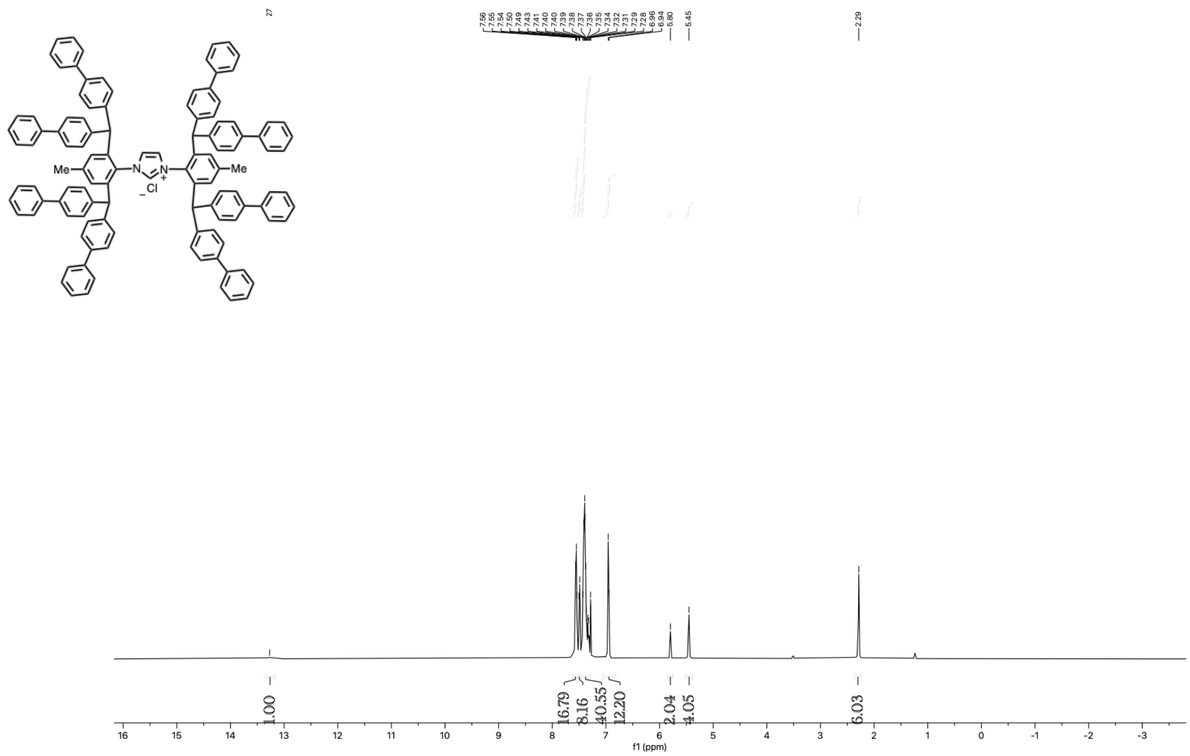

Figure S10 ^{1}H NMR (500 MHz, $CDCl_3$) Spectrum of **5**

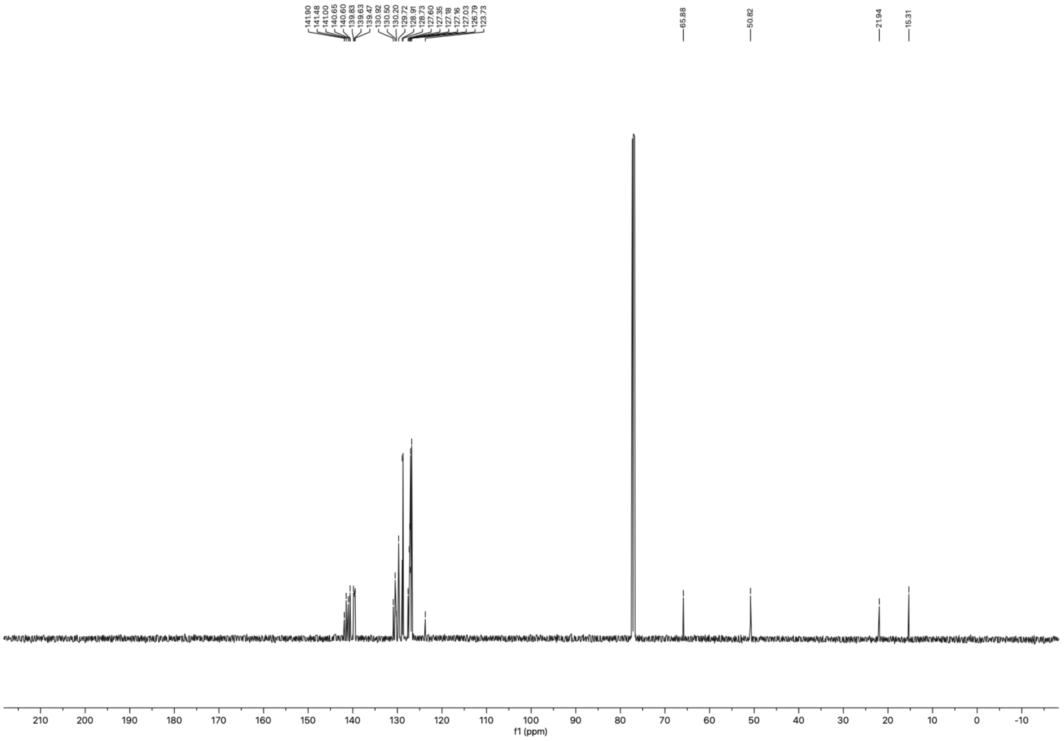

Figure S11 ^{13}C {[1] H} NMR (125 MHz, $CDCl_3$) Spectrum of **5**

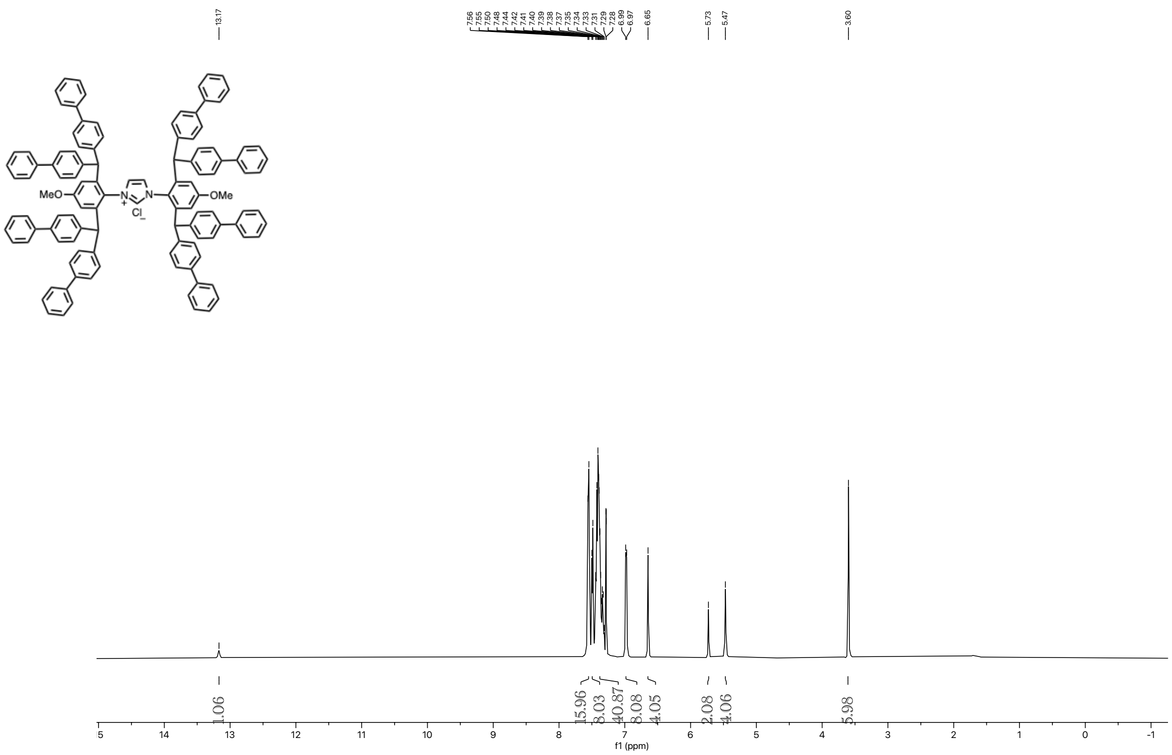

Figure S12 ^{1}H NMR (500 MHz, $CDCl_3$) Spectrum of **6**

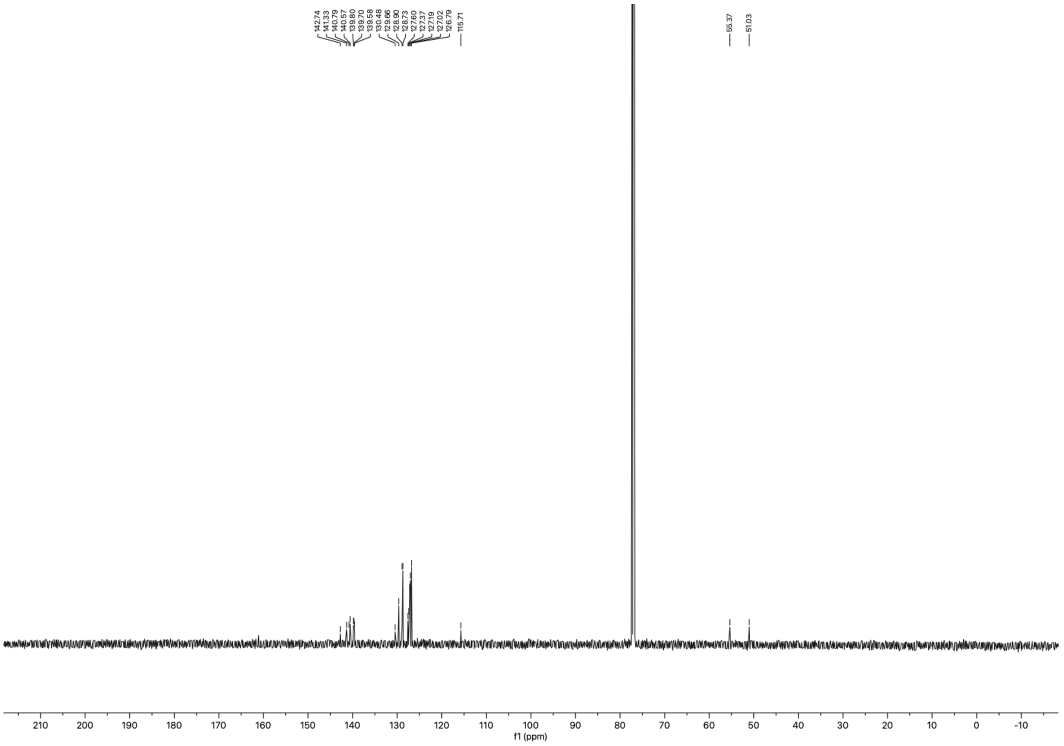

Figure S13 ^{13}C {^{1}H} NMR (125 MHz, $CDCl_3$) Spectrum of **6**

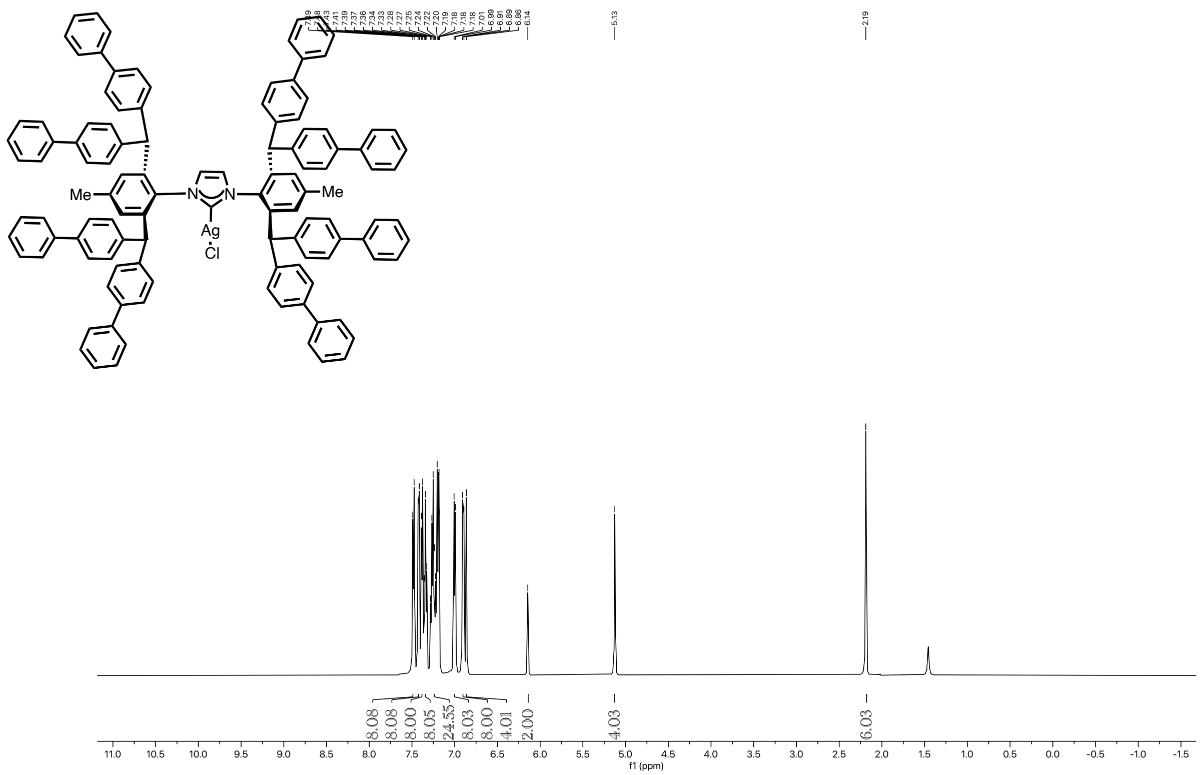

Figure S14 ^{1}H NMR (500 MHz, $CDCl_3$) Spectrum of **7**

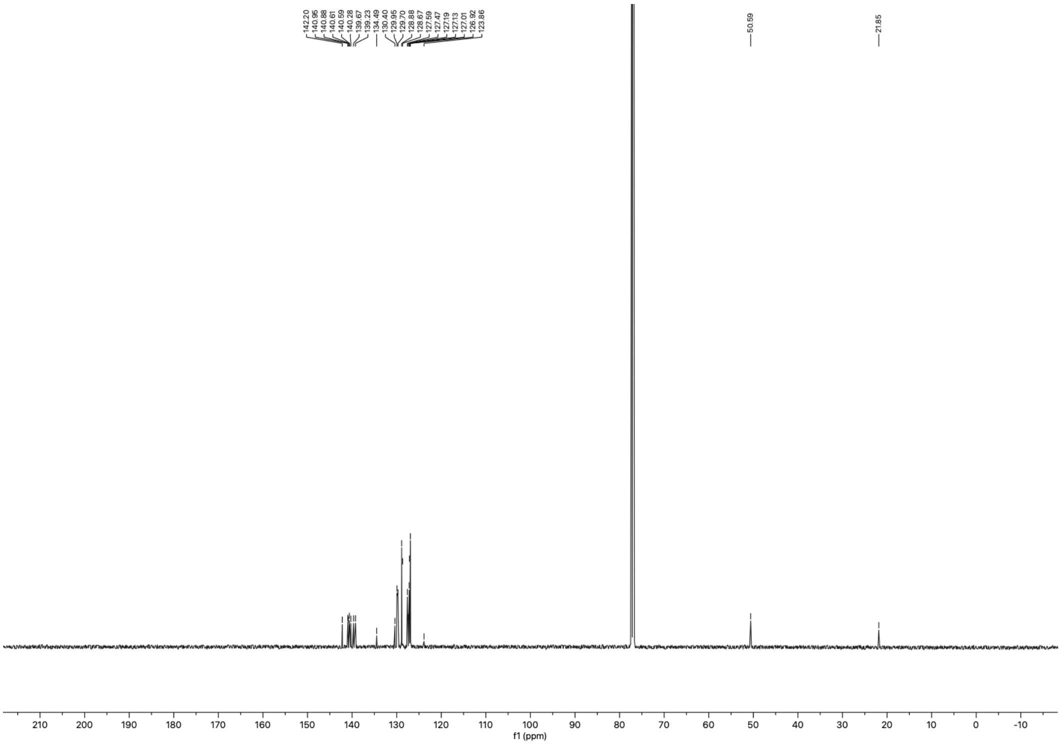

Figure S15 ^{13}C {^{1}H} NMR (125 MHz, $CDCl_3$) Spectrum of **7**

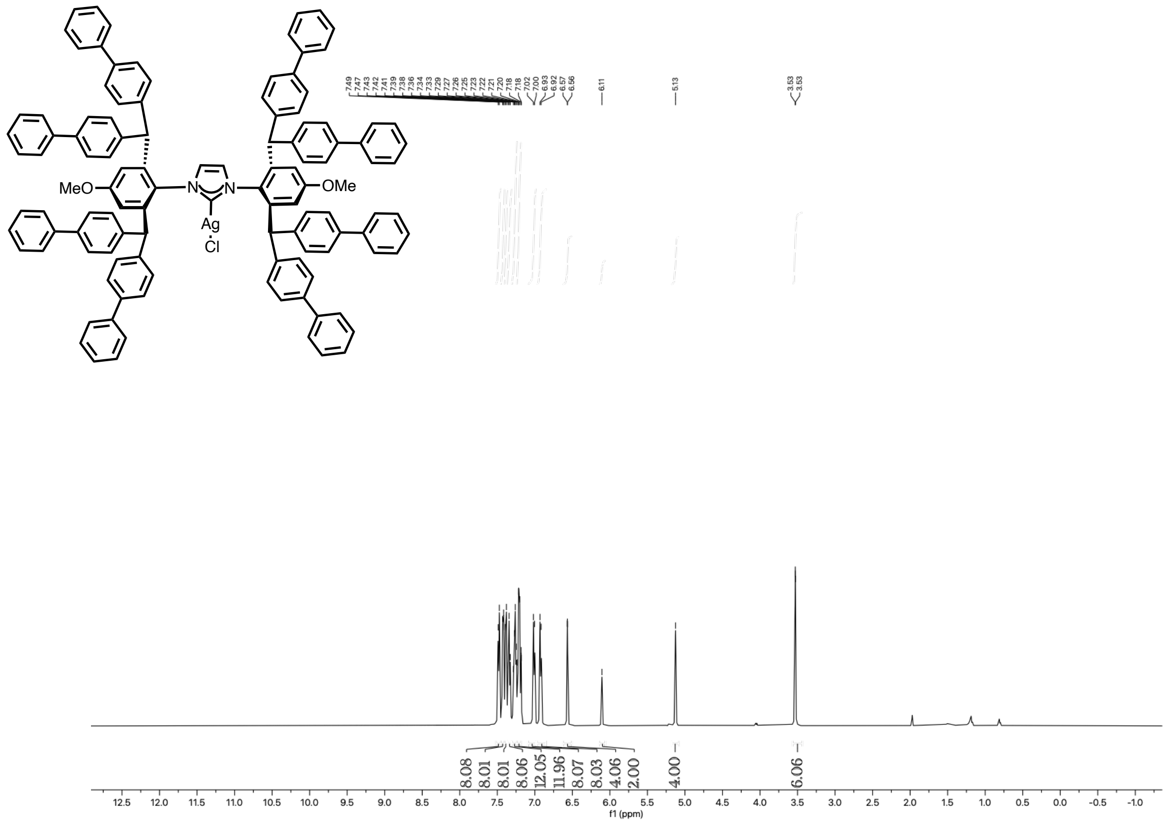

Figure S16 ^{1}H NMR (500 MHz, $CDCl_3$) Spectrum of **8**

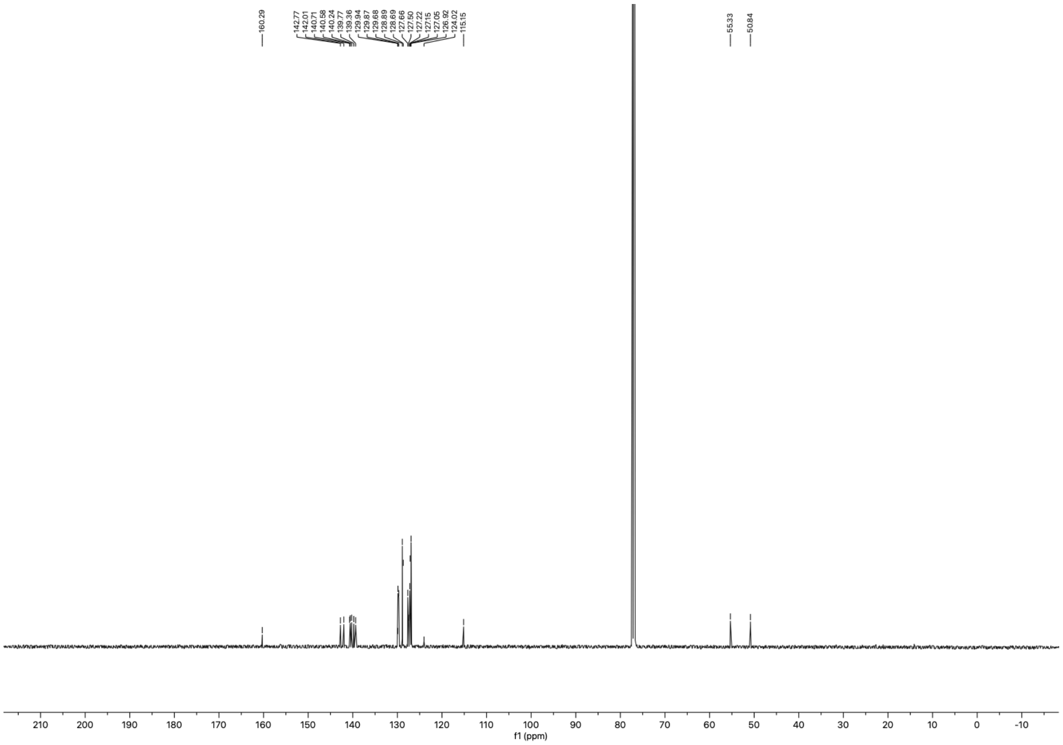

Figure S17 ^{13}C {^{1} H} NMR (125 MHz, $CDCl_3$) Spectrum of **8**

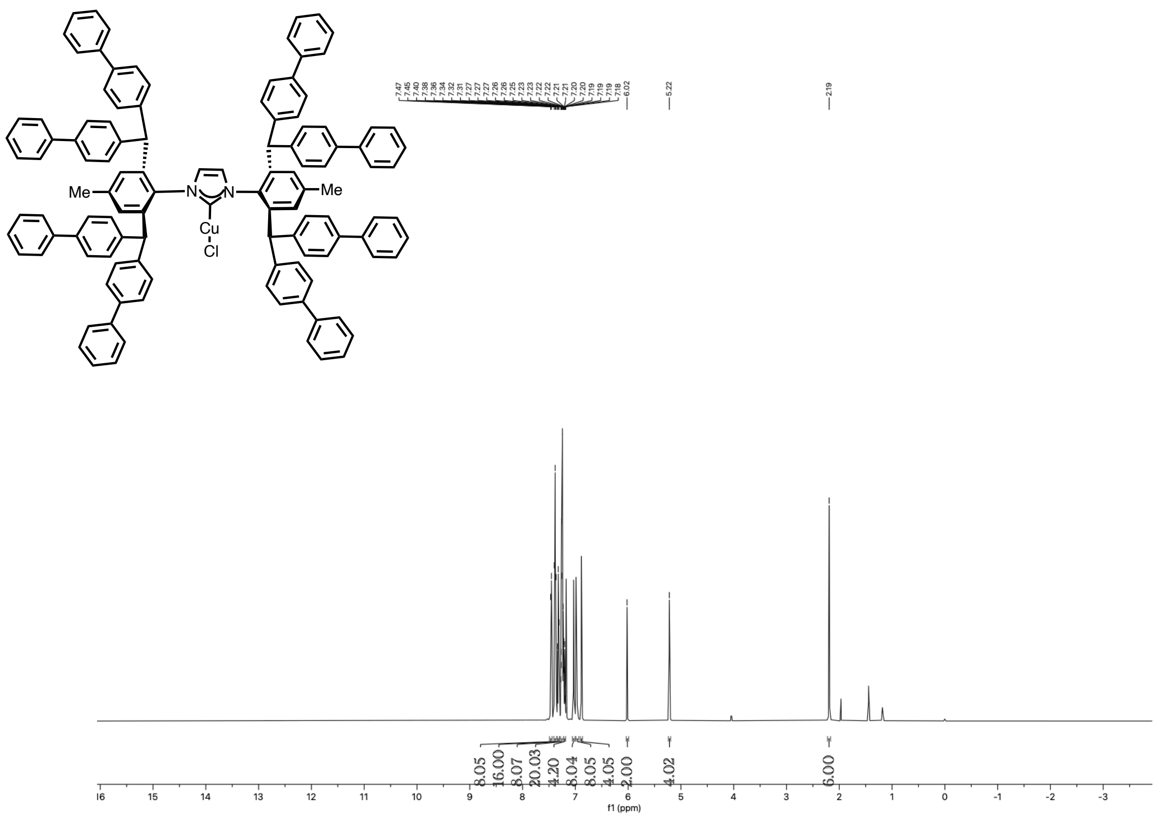

Figure S18 ^{1}H NMR (500 MHz, $CDCl_3$) Spectrum of **9**

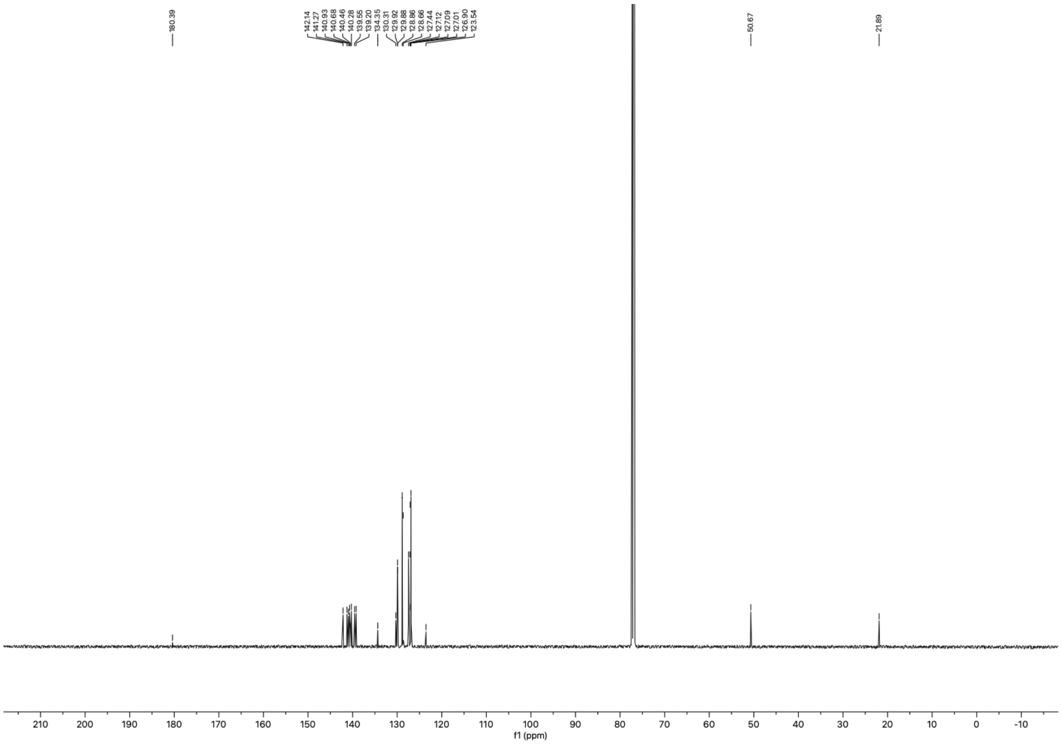

Figure S19 ^{13}C {^{1}H} NMR (125 MHz, $CDCl_3$) Spectrum of **9**

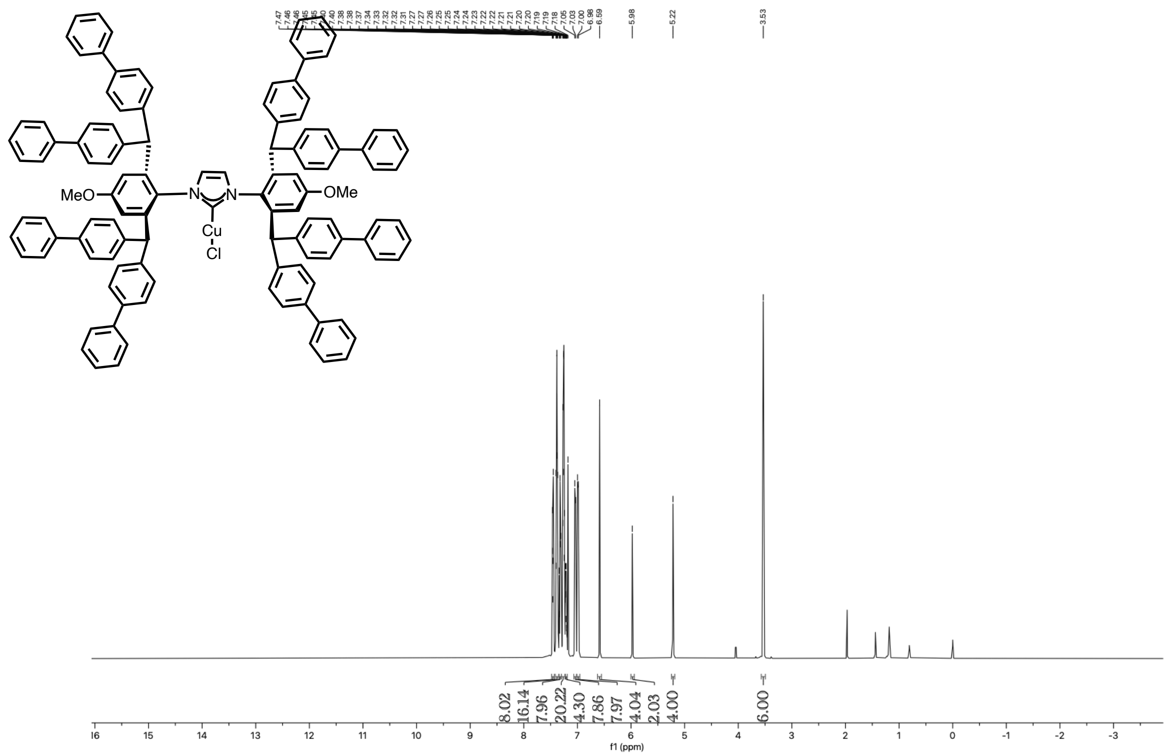

Figure S20 ^{1}H NMR (500 MHz, $CDCl_3$) Spectrum of **10**

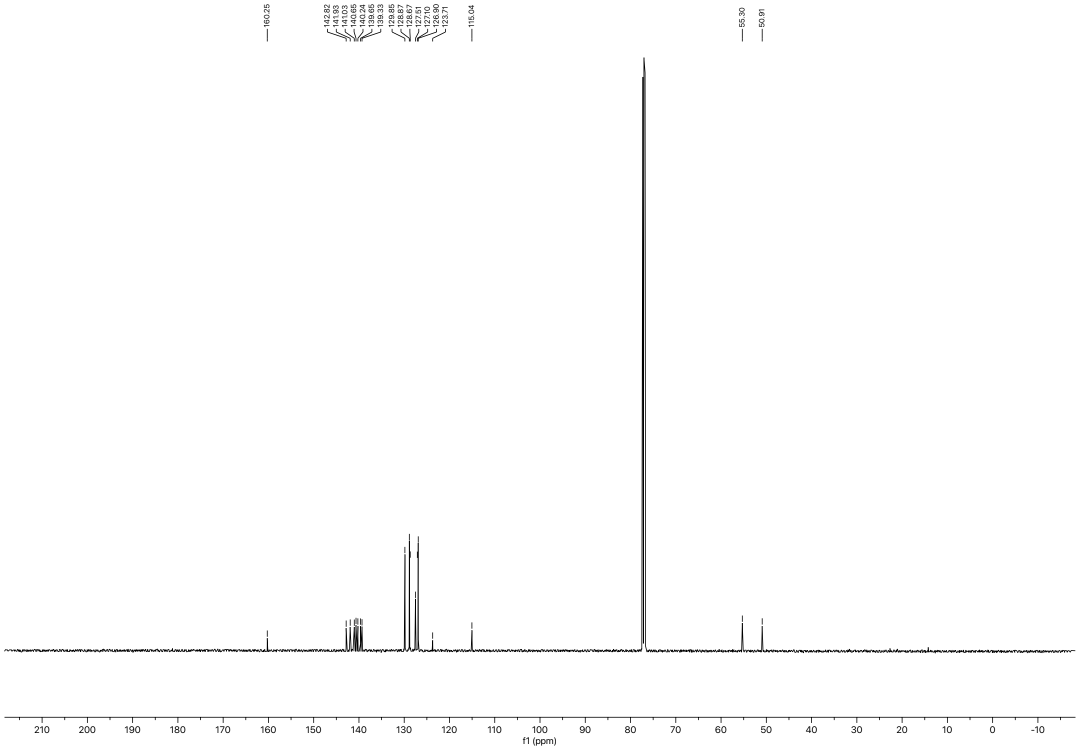

Figure S21 ^{13}C {^{1} H} NMR (125 MHz, $CDCl_3$) Spectrum of **10**

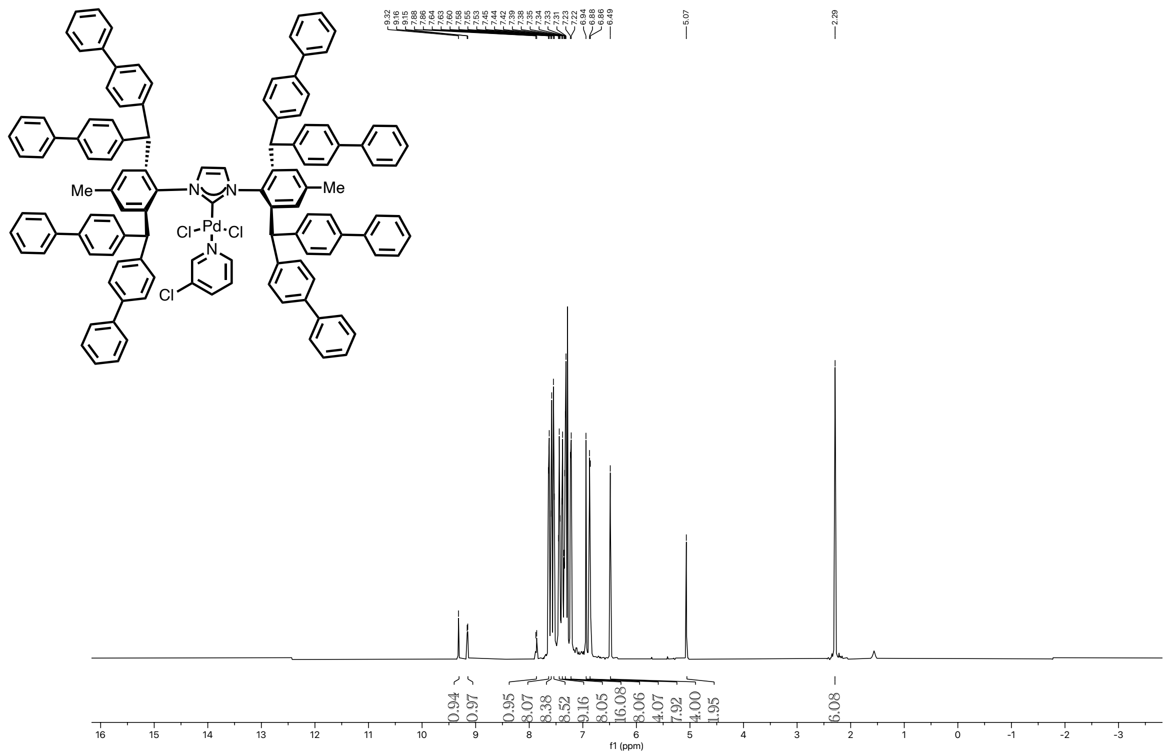

Figure S22 ^{1}H NMR (500 MHz, $CDCl_3$) Spectrum of **11**

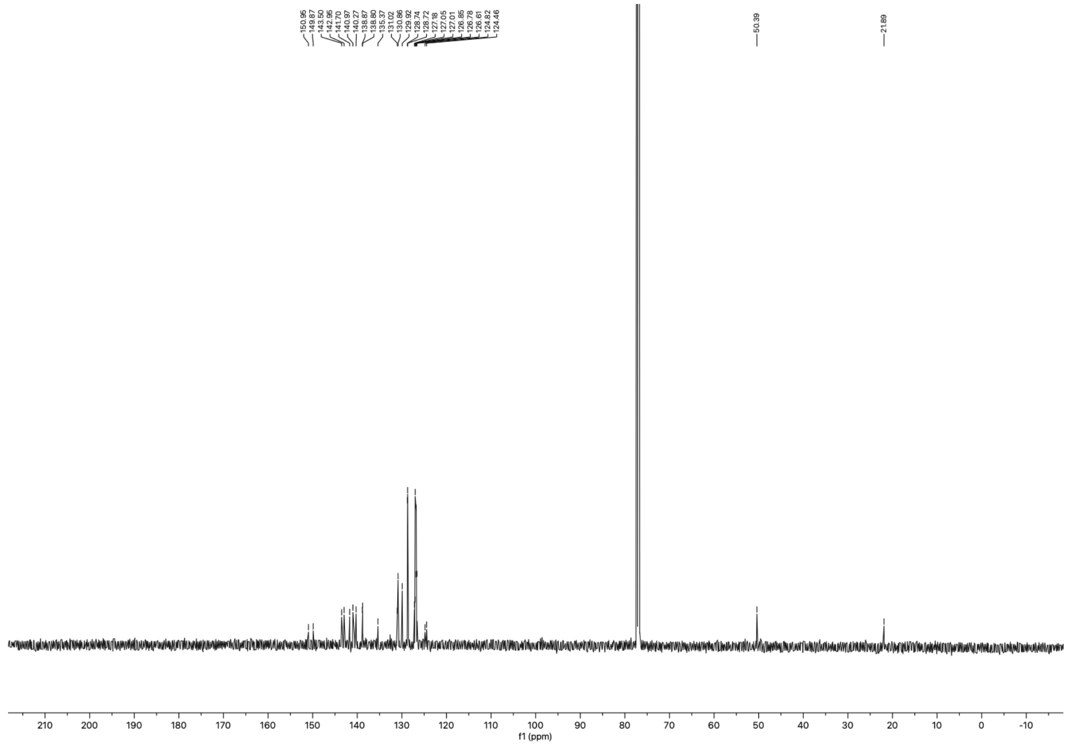

Figure S23 ^{13}C {^{1}H} NMR (125 MHz, $CDCl_3$) Spectrum of **11**

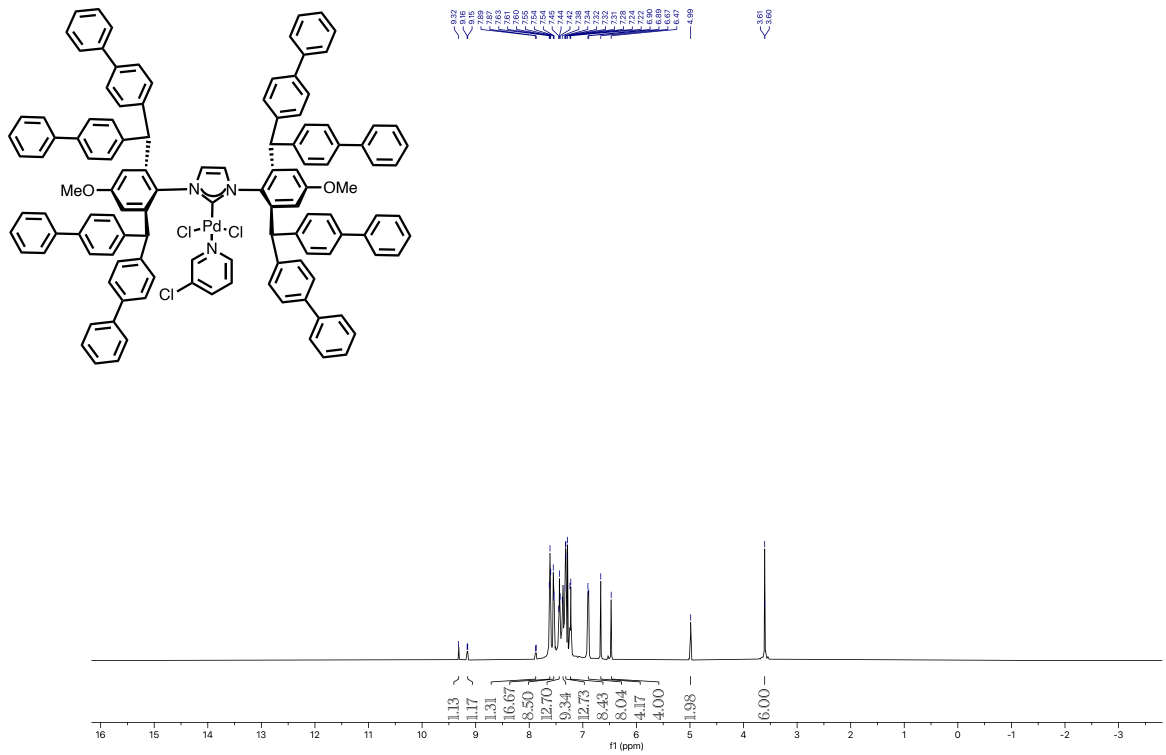

Figure S24 ^{1}H NMR (500 MHz, $CDCl_3$) Spectrum of **12**

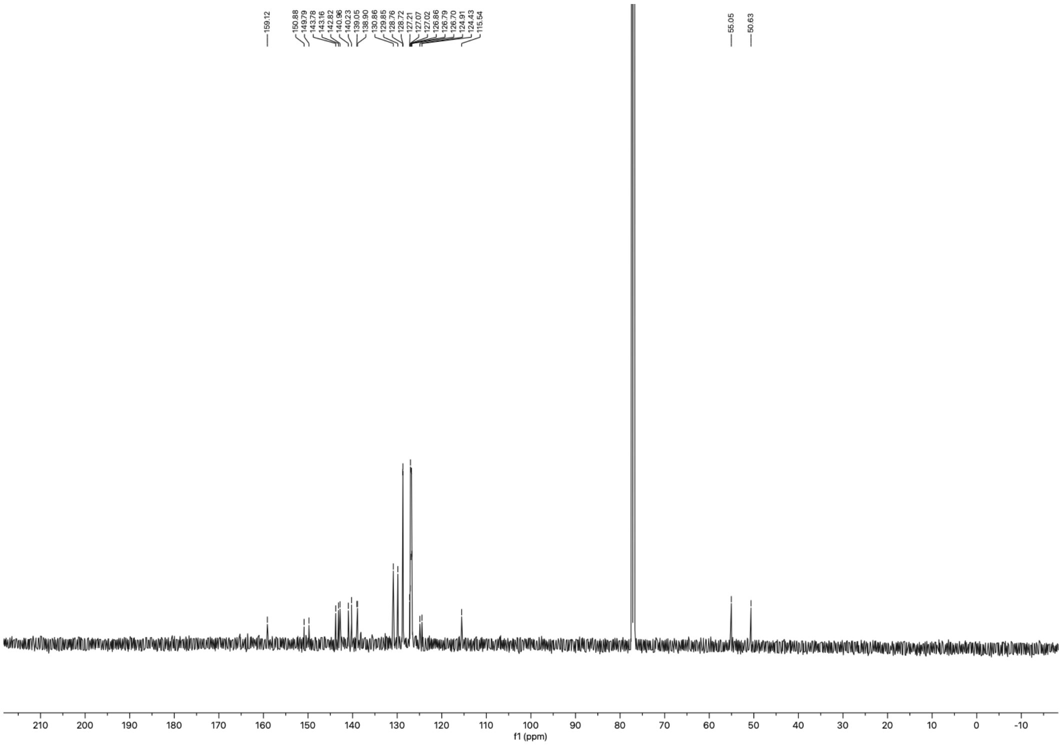

Figure S25 ^{13}C {^{1}H} NMR (125 MHz, $CDCl_3$) Spectrum of **12**

References

[1] Park B.S., Lee S.W., Kim I.T., Tae J.S., Lee S.H. Synthesis and photoluminescent properties of new ceramidine derivatives. *Heteroat. Chem.* **2012**, 23, 66-73.

[2] Guo L., Gao H., Guan Q., Hu H., Deng J., Liu J., Liu F., Wu Q., Substituent effects of the backbone in α-diimine palladium catalysts on homo-and copolymerization of ethylene with methyl acrylate. *Organometallics* **2012**, 31, 6054-6062
